# Near-Room-Temperature Magnetoelectric Coupling Engineered through Inversion-Breaking Tilts in a Bulk Perovskite Polytype

**DOI:** 10.1021/jacs.6c11283

**Published:** 2026-08-27

**Authors:** Struan Simpson, Urmimala Dey, Martin R. Lees, Ivan Da Silva, Nicholas C. Bristowe, Mark S. Senn

**Affiliations:** † Department of Chemistry, 2707 University of Warwick, Gibbet Hill, Coventry CV4 7AL, U.K.; ‡ Luxembourg Institute of Science and Technology, Avenue des Hauts-Fourneaux 5, L4362 Esch-sur-Alzette, Luxembourg; § Department of Physics, University of Warwick, Gibbet Hill, Coventry CV4 7AL, U.K.; ∥ ISIS Facility, Rutherford Appleton Laboratory, Harwell Oxford, Didcot OX11 0QX, U.K.; ⊥ Centre for Materials Physics, 3057 Durham University, South Road, Durham DH1 3LE, U.K.

## Abstract

Systematic strategies to design properties such as ferroelectricity or magnetoelectric coupling are well-established in simple perovskite materials, but they remain scarce in more complex framework structures. Using a hexagonal polytype of the ternary Manganite AMnO_3_ (A= Ba, Sr, Ca) as a model system, we introduce a symmetry-guided design principle in which an inversion-breaking rigid-unit mode (RUM) serves as a single structural instability generating both polar and ferromagnetic orders within a bulk antiferromagnetic material. Symmetry analysis and first-principles calculations reveal that cooperative tilts of the Mn_2_O_9_ bioctahedral dimers generate a spontaneous polarization, and in the antiferromagnetically ordered state, they also induce a ferromagnetic moment. High-resolution diffraction and magnetic susceptibility measurements show that the structural and magnetic orders persist as high as 450 and 280 K, respectively, highlighting the untapped potential of framework structures that deviate from simple perovskite motifs to be designed to host useful ferroic properties. Our approach establishes a transferable symmetry-based framework for engineering ferroelectric and magnetoelectric states across chemically diverse framework architectures.

## Graphical abstract

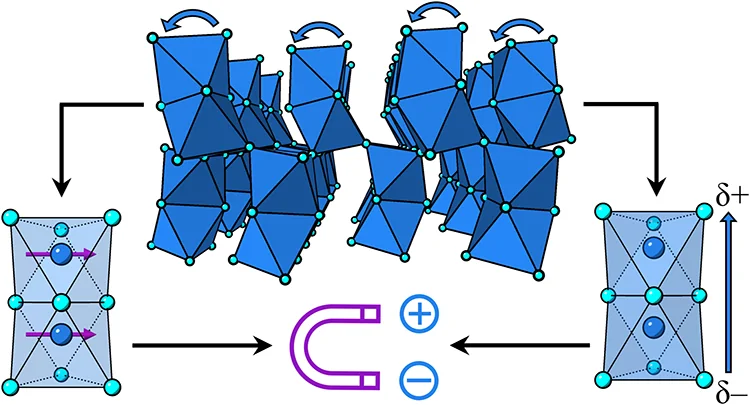

## Introduction

The rapid expansion of artificial intelligence has intensified the demand for energy-efficient memory and logic technologies. Addressing this demand requires materials that enable low-power control of magnetic states, motivating renewed interest in magnetoelectric (ME) multiferroics in which magnetic order can be manipulated via applied electric fields.^1^ Prototype ME switching devices have demonstrated substantial improvements in endurance and energy efficiency compared to existing devices.^2^ Nevertheless, materials with robust ME coupling near room temperature remain exceptionally rare. This scarcity stems from the longstanding challenge of combining the chemical ingredients required for large ferroelectric (FE) polarizations (e.g., d^0^ cations such as Ti^4+^) and ferromagnetism (i.e., cations with unpaired electrons) within the same material.^3^ The rational design of new ME materials represents an enduring challenge in modern materials chemistry.

Recently, substantial progress has been made in the design of new ME materials through group-theoretical methods. The fundamental principle of this approach is to identify a material system in which a ferroelectric (FE) structural distortion and weak ferromagnetism (wFM) derive from a common symmetry-breaking origin, enabling both properties to be controlled via an intermediary distortion. This principle was first elucidated in the form of the hybrid-improper ferroelectric mechanism, where two or more nonpolar octahedral tilt modes may be combined to generate and control a spontaneous polarization.^4^ This was first demonstrated in PbTiO_3_/SrTiO_3_ superlattices^5^ before being extended to a swathe of layered perovskite materials such as A_3_B_2_O_7_ Ruddlesden–Popper phases^6–8^ and AA′B_2_O_7_ Dion–Jacobson phases.^9,10^ Importantly, this purely geometric source of ferroelectricity is compatible with the presence of unpaired electrons on the B-site, enabling the coexistence of polar and magnetic orders within the same material. This approach has since been extended to enumerate robust mechanisms of ME coupling in other perovskite-based materials, where specific combinations of symmetry-breaking distortions can be designed that yield directly coupled FE and wFM orders.^11–13^ Nevertheless, magnetic ordering in ME perovskite-based materials is often limited to cryogenic temperatures.^14–18^ This has inevitably limited the practicality of these systems for functional applications, spurring ongoing efforts to discover new ME materials with more practical operating temperatures.

One possible workaround is to apply group-theoretical approaches to structures that deviate from simple corner-sharing connectivity between their polyhedra. Frameworks incorporating edge- or face-sharing polyhedra offer alternative exchange pathways characterized by strong orbital overlap between nearest-neighbor metal sites, hence their enhanced exchange interactions could lead to higher magnetic ordering temperatures.^19–22^ However, these frameworks are generally thought to be less flexible than those composed of corner-sharing polyhedra,^23^ so they are often assumed to lack practical structural instabilities that can be readily induced to achieve coupled polar and magnetic orders. Indeed, this has represented the key bottleneck inhibiting the group-theoretical design of ferroic functionalities in framework structures that deviate from conventional perovskite motifs.

Here, we exploit a newly discovered rigid-unit mode (RUM) in a four-layer hexagonal (4H) polytype of the perovskite structure (Figure 1a) to induce mutually coupled polar and wFM orders close to room temperature (∼280 K, ∼7 °C). Perovskite polytypes (sometimes referred to as “hexagonal perovskites”) are generally characterized by face-sharing connectivity between their octahedra, hence they provide an ideal platform to identify new forms of structural instability that could yield useful functionalities in more complex framework topologies. Using 4H-AMnO_3_ (A= Ba^2+^, Sr^2+^, Ca^2+^) as a model system (Figure 1b), we use high-resolution diffraction, first-principles calculations, and magnetic susceptibility measurements to show how a single inversion-breaking RUM can be engineered in this system to induce the mutually coupled polar and wFM orders required for ME coupling. In contrast to established tilt-driven mechanisms in perovskite-based materials,^11–13^ the mechanism we identify here is remarkably simple, requiring minimal symmetry-breaking distortions to fulfill the necessary chemical ingredients for ME coupling. More generally, our insights showcase the potential of stabilizing inversion-breaking RUMs in more complex framework topologies to confer them with newfound functionality.

**Figure 1:**
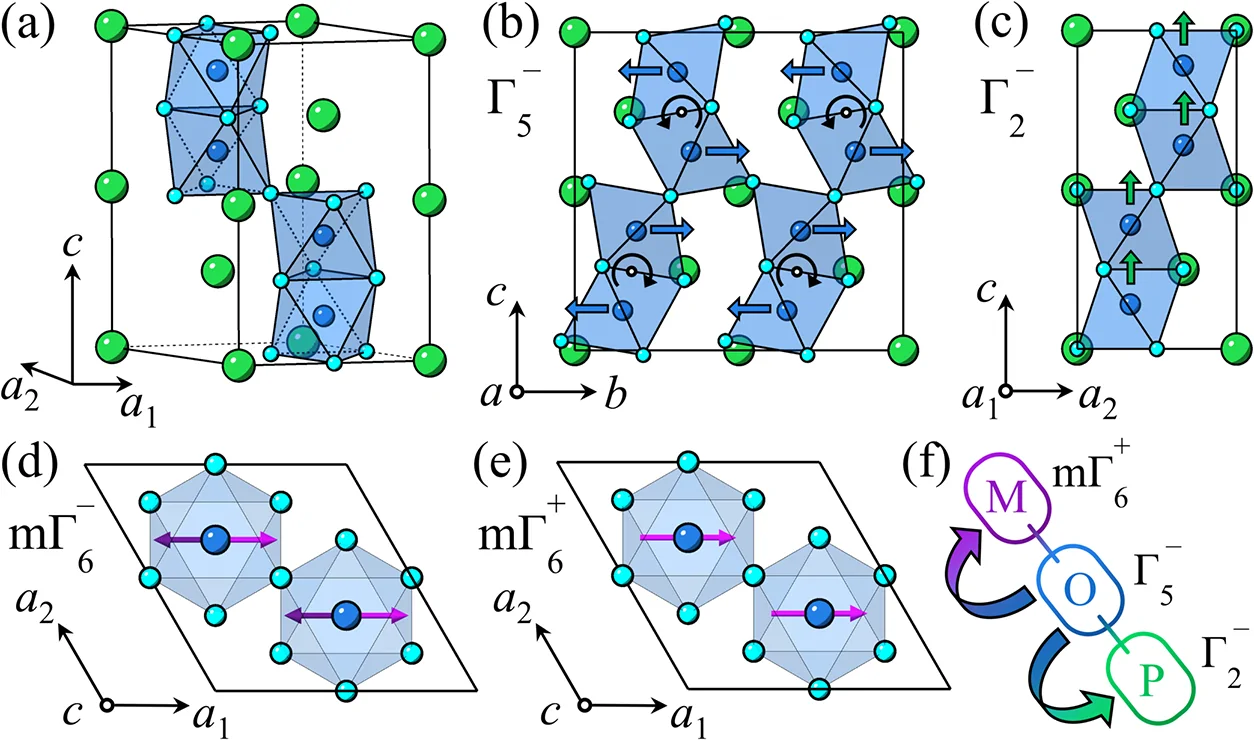
Symmetry-breaking distortions in 4H-SrMnO_3_. (a) The *P*6_3_/*mmc* aristotype structure, with green, blue, and cyan atoms representing the Sr, Mn, and O sites, respectively. (b) The room-temperature *Cmc*2_1_ structure, highlighting the Γ_5_^–^ tilt mode of the Mn_2_O_9_ bioctahedra (black arrows) and the accompanying antipolar Mn displacements (blue arrows). (c) The Γ_2_^–^ polar distortion depicted with respect to the parent structure; only the Mn displacements (green arrows) have been shown for visual clarity. (d), (e) The AFM and wFM spin components, transforming as mΓ_6_^–^ and mΓ_6_^+^, respectively. For the mΓ_6_^–^ AFM component, light and dark purple arrows map to the Mn sites at *z* ≈ 3/8, 7/8 and *z* ≈ 1/8, 5/8, respectively. (f) The fundamental design principle yielding ME coupling in 4H-SrMnO_3_, showing how octahedral tilting (O) simultaneously induces both an electrical polarization (P) and a spontaneous magnetization (M).

## Experimental and Theoretical Methods

First-principles calculations were performed within the density functional theory (DFT) framework as implemented in the Vienna Ab initio Simulation Package (VASP), version 6.4.3.^24^ Projector augmented-wave (PAW) pseudopotentials (PBE, version 5.4)^25^ were used with the following valence electron configurations: 4s^2^4p^6^5s^2^ (Sr), 3p^6^3d^5^4s^2^ (Mn), and 2s^2^2p^4^ (O). The PBEsol flavor of general gradient approximation (GGA) exchange–correlation functional^26^ was employed to accurately describe the structural properties of bulk 4H-SrMnO_3_. The energy convergence criterion was set at 10^–9^ eV for all calculations, and full structural relaxations were carried out until the Hellmann–Feynman forces on each atom were less than 1 meV/Å. Correlation effects were treated within the GGA + *U* formalism introduced by Dudarev et al.^27^ using an effective on-site Hubbard parameter of *U*_eff_ = 4.5 eV for the Mn 3d orbitals.^28,29^ We varied the *U*_eff_ values within a reasonable range to examine the stability of the ground-state magnetic order. We performed convergence tests for the *P*6_3_/*mmc* aristotype structure with the ground-state A-type antiferromagnetic (AFM) spin configuration and found that a 700 eV plane wave energy cutoff and a 5 × 5 x 3 ***k***-point mesh were sufficient to converge the energy differences, atomic forces, and stresses within 0.01 meV/f.u., 0.2 meV/Å, and 0.2 GPa, respectively. Spin–orbit coupling (SOC) effects were included self-consistently in the noncollinear calculations. The Berry phase method^30,31^ within VASP is used to compute spontaneous electric polarization. Phonon calculations were performed using the finite displacement method for a 2 × 2 x 2 phonon supercell, and phonon dispersions were generated with PHONOPY.^32^ The ISOTROPY software suite was employed for subsequent symmetry analysis.^33^

Pristine polycrystalline samples of 4H-AMnO_3_ (A = Ba^2+^, Sr^2+^, Ca^2+^) were synthesized according to a previous method.^34^ Stoichiometric quantities of BaCO_3_ (Sigma-Aldrich, ≥99.9%), SrCO_3_ (Sigma-Aldrich, ≥ 99.9%), CaCO_3_ (Sigma-Aldrich, ≥99.9%), and MnO_2_ (Sigma-Aldrich, ≥99%) were mixed and ground using an agate mortar and pestle before being pressed into 10 mm pellets and heated to 900 °C in an alumina crucible for 10 h. The pellets were then reground and reheated twice for 10 h at 1250 °C to ensure both high purity and crystallinity. Due to the tendency for SrMnO_3_ to reduce above ∼1050 °C,^34^ each sample was also subjected to an additional 24 h dwell at 600 °C to ensure that the nominal oxygen stoichiometry was accurate. This was later corroborated by Rietveld refinements against neutron powder diffraction (NPD) data, which showed that the *A*, Mn, and O sites could each be modeled to within ±1% of their nominal occupancies. Laboratory X-ray diffraction (XRD) measurements showed that the samples prepared this way typically contained only trace impurities of a Mn_3_O_4_ spinel phase (<0.5 wt %). An oxygen-deficient sample of the form SrMnO_3−δ_ was also prepared by heating oxygen-stoichiometric SrMnO_3_ to 1250 °C for 24 h before quenching the sample in air; further details are provided in the Supporting Information (Supporting Note 7).

High-resolution synchrotron XRD (S-XRD) measurements^35^ were performed at the ID22 beamline (ESRF, France) using a 35 keV X-ray beam and a 13-channel Si(111) multianalyzer stage. Samples of 4H–Sr_1–*x*_Ca_*x*_MnO_3_ (*x* = 0, 0.05, and 0.10) were loaded into a 0.5 mm borosilicate capillary before being measured in the temperature range of 10–500 K for ∼45 min at each temperature point. Additional S-XRD measurements were performed for 4H-SrMnO_3_ at the I11 beamline (Diamond Light Source, U.K.) using a 15 keV X-ray beam. The sample was loaded into a 0.3 mm quartz capillary to minimize absorption effects, and data were collected continuously in the ranges of 100–300 K and 25–300 °C using a position-sensitive Mythen detector. NPD measurements were performed on the GEM diffractometer (ISIS Neutron and Muon Source, U.K.).^36^ ∼1.5 g of 4H-SrMnO_3_ were loaded into a vanadium can for these measurements. Data were collected for ∼3 h at 10 K, but due to time limitations, data were collected for ∼1.5 h at 290 K. Symmetry-adapted Rietveld fits were performed with Topas using the jEdit interface.^37,38^ Full crystallographic data from the refinements have been provided in the Supporting Information. Additional symmetry analysis was performed using the ISODISTORT,^39,40^ INVARIANTS,^41,42^ and ISOTILT^43,44^ webtools. Mode amplitudes have been normalized with respect to the parent cell, but note that since all of the symmetry-breaking distortions transform as Γ-point irreducible representations (irreps), these are the same as the subcell-normalized amplitudes.

DC magnetic susceptibility data were collected on 4H-BaMnO_3_ and 4H–Sr_1–*x*_Ca_*x*_MnO_3_ using a Quantum Design Magnetic Properties Measurement System (MPMS). Zero-field-cooled (ZFC) and field-cooled (FC) data were collected between 5 and 300 K using a 1000 Oe field. Hysteresis loops were recorded at various temperatures between 200 and 300 K and fields between −50 and 50 kOe. DC resistivity data were collected in the range of 250–400 K with a Quantum Design Physical Property Measurement System (PPMS). A four-probe method was used to measure polycrystalline bars with typical dimensions of 5 mm × 3 mm × 1 mm.

## Theoretical Basis

The basis of our design philosophy is informed by our recent study of an exotic polar instability in the six-layer hexagonal (6H) polytype of the canonical ferroelectric perovskite BaTiO_3_,^45^ which provides a prototype for the symmetry-coupled polar distortions we seek to generalize here. In 6H-BaTiO_3_, FE order emerges due to a second-order Jahn–Teller (SOJT) distortion associated with the Ti^4+^ (d^0^) cations. This distortion primarily manifests in terms of antipolar displacements of the Ti^4+^ cations within the hexagonal basal plane, but because the TiO_6_ octahedral faces are tilted by ∼9° out of the plane, an additional polar component is induced along the *c*-axis to ensure the Ti^4+^ cations displace toward the octahedral face centers. Group-representational symmetry-mode analysis shows the coupled antipolar and polar displacements transform with respect to the *P*6_3_/*mmc* aristotype structure as the Γ_5_^–^ and Γ_2_^–^ irreps, respectively. We further predicted that analogous distortions could emerge in several related polytypes (i.e., 4H, 8H, and 12H) that share the same *P*6_3_/*mmc* aristotype symmetry. However, so far, only the 6H polytype of BaTiO_3_ has been experimentally realized. Additionally, the polarization is contingent on the SOJT distortion of the Ti^4+^ cations, so this mechanism precludes coupling to magnetic order. These limitations motivated us to search for alternative structural instabilities that could yield analogous polarization coupling while remaining chemically compatible with magnetism.

Recently, we predicted a class of inversion-breaking RUMs^46^ in several perovskite polytypes that are capable of inducing a spontaneous polarization on an improper basis. Formally, RUMs represent low-energy phonon modes in framework materials that do not distort the internal bonding environments of the coordination polyhedra,^47^ hence they are expected to be lower in energy than all of the other vibrational modes of the framework. RUMs thus represent feasible structural deformations that are the most likely to have sufficiently low frequencies to “freeze out” with respect to the undistorted parent structure. For example, in conventional (3C) *ABX*_3_ perovskites, RUMs are more familiarly known in terms of cooperative tilts or rotations of the *BX*_6_ octahedra, which can be frozen out with respect to the *Pm*3̅*m* aristotype structure to yield lower-symmetry structures.^48,49^ These tilts can be controlled by varying the degree of size mismatch between the average radii of the A- and B-site cations,^50^ and this represents a more general strategy by which RUMs may be manipulated to induce phase transitions in other materials as well.

Beyond their initial significance in characterizing distorted structures, RUMs have important implications for the material functionality. In particular, they are foundational to the mechanisms of hybrid improper ferroelectricity and ME coupling which have been elucidated in perovskite-based materials.^4,12^ In contrast, RUMs in edge- or face-sharing polyhedral frameworks have historically been considered unfavorable because of the higher number of geometric constraints imposed upon the framework, inhibiting their flexibility.^49^ This is precisely what has previously prohibited the scope for the rational design of functional properties in more complex framework structures. However, our recent symmetry insights into perovskite polytype structures have overturned this longstanding consensus: several polytypes host RUMs involving intrinsic antipolar displacements of the *B*-site cations,^46^ thereby breaking inversion symmetry directly without the need for extraneous symmetry-breaking distortions (e.g., cation/anion ordering or SOJT distortions). This contrasts with 3C perovskites, where additional symmetry-breaking mechanisms such as cation order or SOJT distortions are required to couple to the octahedral tilts to induce FE order.^12^ Consequently, hexagonal polytypes offer appealing advantages compared to 3C perovskites as a platform for designing polar or ME functionalities.

To demonstrate this design principle, we consider the 4H perovskite polytype (Figure 1a). In this structure type, the Γ_5_^–^ RUM corresponds to an in-phase tilting of the B_2_X_9_ bioctahedra about an axis lying in the hexagonal basal plane (Figure S1). Condensing the action of the associated order parameter on the high-symmetry parent phase yields a series of structures with either *C*222_1_, *Cmc*2_1_, or *P*2_1_ symmetry, depending on the orientation of the tilt axis (i.e., order parameter direction). While all three structures belong to piezoelectric point groups, the *Cmc*2_1_ (Figure 1b) and *P*2_1_ structures (Figure S1c) are also polar point groups (*mm*2 and 2, respectively); therefore, they may display a spontaneous polarization. Microscopically, this is due to a series of out-of-plane displacements of the A, B, and X atoms transforming as the Γ_2_^–^ irrep (Figure 1c), demonstrating the same polarization coupling that we identified in 6H-BaTiO_3_.^45^ By analogy to how octahedral tilt modes are stabilized in 3C perovskites,^50^ *A*/*B*-site size mismatch is expected to condense the Γ_5_^–^ RUM, thus inducing static polyhedral tilting and a spontaneous polarization. Crucially, this RUM-based mechanism is fully compatible with the presence of magnetic *B*-site cations, providing a viable route to combine FE and wFM orders within a single phase. In the following, we demonstrate the experimental realization of our design strategy.

## Results and Discussion

To demonstrate how FE order may be induced via the Γ_5_^–^ tilt mode, we consider the ternary perovskite system 4H-*A*MnO_3_ (A= Ba^2+^, Sr^2+^, Ca^2+^). The magnetic Mn^4+^ (d^3^) cations make this system an ideal platform to assess the compatibility of the induced FE and magnetic orders, and this system is generally characterized by relatively high Néel temperatures (*T*_N_ ≈ 263 and 280 K for A = Ba^2+^ and Sr^2+^, respectively^51,52^ ). We begin by showing that the necessary symmetry conditions required for FE order and a wFM moment—namely, the Γ_5_^–^ tilts—can be stabilized in this system. For A = Ba^2+^, the crystal structure retains the aristotype *P*6_3_/*mmc* symmetry down to at least 5 K;^53^ for A= Sr^2+^, a structural phase transition was previously reported to occur below ∼350 K.^52^ The low-symmetry 4H-SrMnO_3_ structure was previously assigned to the noncentrosymmetric (but nonpolar) *C*222_1_ space group, which is consistent with the predictions of our RUM analysis. However, our high-resolution S-XRD data (Figure 2a) reveal that this model provides a poor description of the observed superstructure reflections (Figures S2 and S3), and Rietveld refinements yielded consistently worse fitting statistics compared to the alternative *Cmc*2_1_ or *P*2_1_ models (Table S1). Furthermore, variable-temperature S-XRD measurements (Figure 2b) indicate that the structural transition temperature (*T*_s_) is closer to 450 K, suggesting previous studies underestimated *T*_S_ by ∼100 K. These discrepancies motivated us to reinvestigate the structure of 4H-SrMnO_3_ to determine the correct tilt orientation and establish the possibility of a RUM-induced FE order.

**Figure 2:**
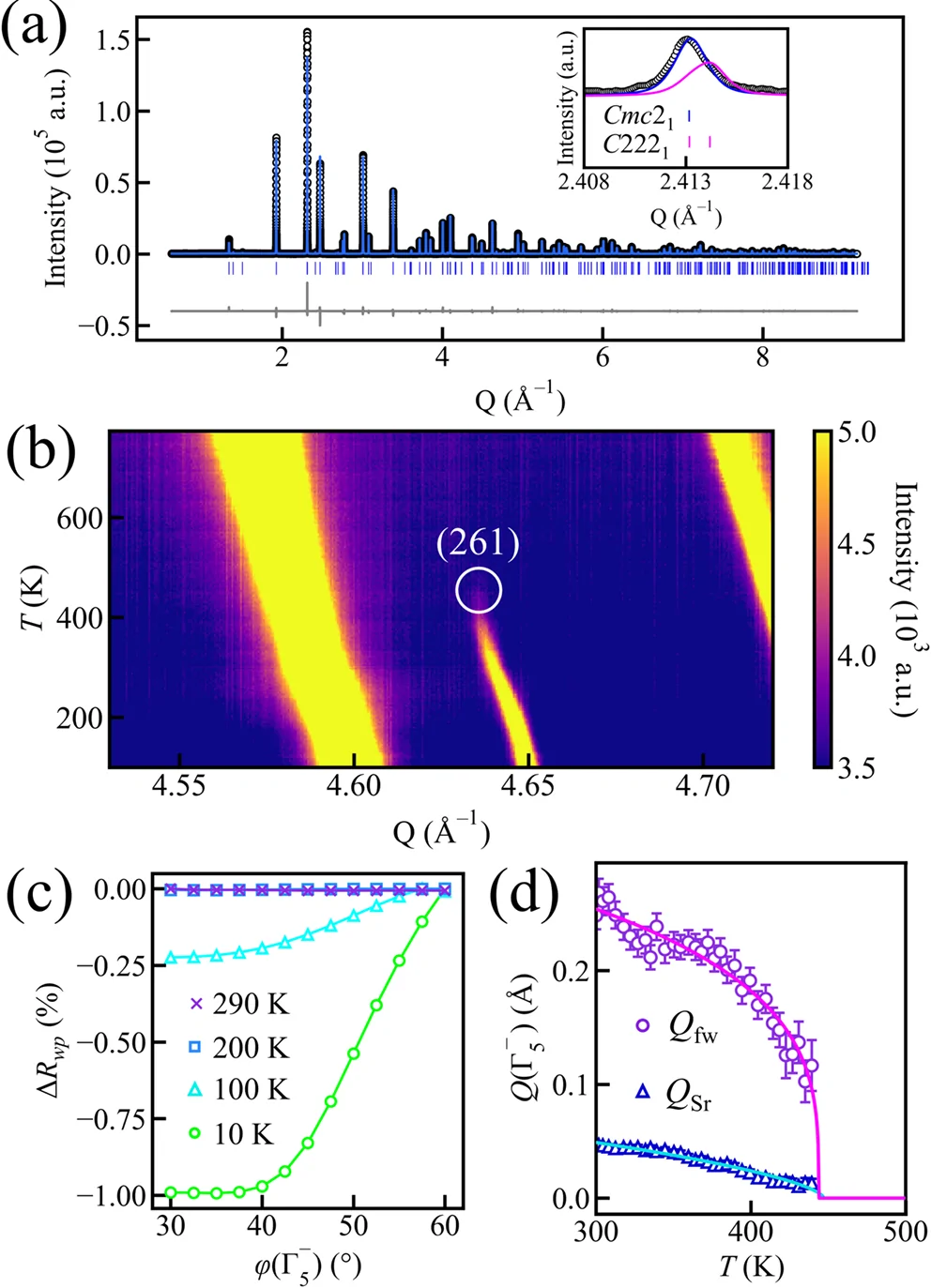
Crystallographic analysis of 4H-SrMnO_3_. (a) Rietveld fit of the *Cmc*2_1_ structural model against high-resolution S-XRD data collected at 10 K on the ID22 beamline (λ = 0.3544921(1) Å). The inset compares the fits of the *C*222_1_ (bottom ticks) and *Cmc*2_1_ (top ticks) models for the (131) superstructure reflection (indexed with respect to the *Cmc*2_1_ structure). (b) Heat map of variable-temperature S-XRD data collected on the I11 beamline (λ = 0.8287110(1) Å). The disappearance of the (261) superstructure reflection has been circled. (c) Significance tests based on structural models refined against ID22 data using fixed increments of the Γ_5_^–^ tilt axis (φ­(Γ_5_^–^)) below *T*_s_. φ = 30° and 60° correspond to *Cmc*2_1_ and *C*222_1_ symmetries, respectively, while intermediate values correspond to *P*2_1_ symmetry. The differences in *R*_wp_ (Δ*R*_wp_) have been calculated with respect to the maximum *R*_wp_ obtained so that Δ*R*_wp_ = 0% for the worst-fitting model. (d) The refined Γ_5_^–^ mode amplitudes decomposed in terms of the framework contribution (*Q*_fw_) and the Sr displacements (*Q*_Sr_). Solid lines depict fits against a power law expression *Q* = *A*(*T*_s_ – *T*)^β^.

Exploiting the strong sensitivity of powder diffraction to lattice strain, we used the coupling between the orthorhombic strain transforming as the Γ_5_^+^ irrep and the Γ_5_^–^ tilt orientation to distinguish between candidate space group assignments (see Supporting Note 2). In short, this is made possible by the fact that the Γ_5_^–^ tilt orientations in the *Cmc*2_1_ and *C*222_1_ phases map to distinct Γ_5_^+^ strain states, allowing the Γ_5_^–^ tilt axis to be inferred from the orientation of the Γ_5_^+^ strain. We performed significance tests with respect to the orientation of the Γ_5_^–^ tilt axis (φ­(Γ_5_^–^)) against our high-resolution S-XRD data collected on ID22 (Figure 2c). At 10 K, we find that a *Cmc*2_1_ structural model (φ = 30°) leads to a better fit (*R*_wp_ = 9.3%) compared to the canonical *C*222_1_ model (φ = 60°; *R*_wp_ = 10.2%), despite both models employing the same number of refined parameters. Additionally, the *C*222_1_ model predicts a subtle splitting of the superstructure reflections (Figure S3), which is not observed within the very high instrumental resolution of our S-XRD measurements (Δ*d*/*d* ≈ 1 × 10^–4^), whereas the *Cmc*2_1_ model accurately reproduces their appearance. This assignment will be further supported by our subsequent DFT calculations, which confirm that the ground-state structure is described by *Cmc*2_1_ symmetry (Figure 3). Although the reduction in ferroelastic strain above 100 K limits our sensitivity to the space group assignment at higher temperatures, the absence of any anomalies in the lattice parameters (Figures S4 and S9) supports a single low-symmetry phase below *T*_S_ ≈ 450 K. Notably, this contrasts with 6H-BaTiO_3_, where a *Cmc*2_1_ → *C*222_1_ transition at *T*_c_ ≈ 70 K is readily distinguished by discontinuities in the lattice parameters.^45^ Therefore, we conclude that 4H-SrMnO_3_ adopts the polar *Cmc*2_1_ structure for all *T* < *T*_S_. Refined structural parameters for the final *Cmc*2_1_ model are provided in Figure S4.

**Figure 3:**
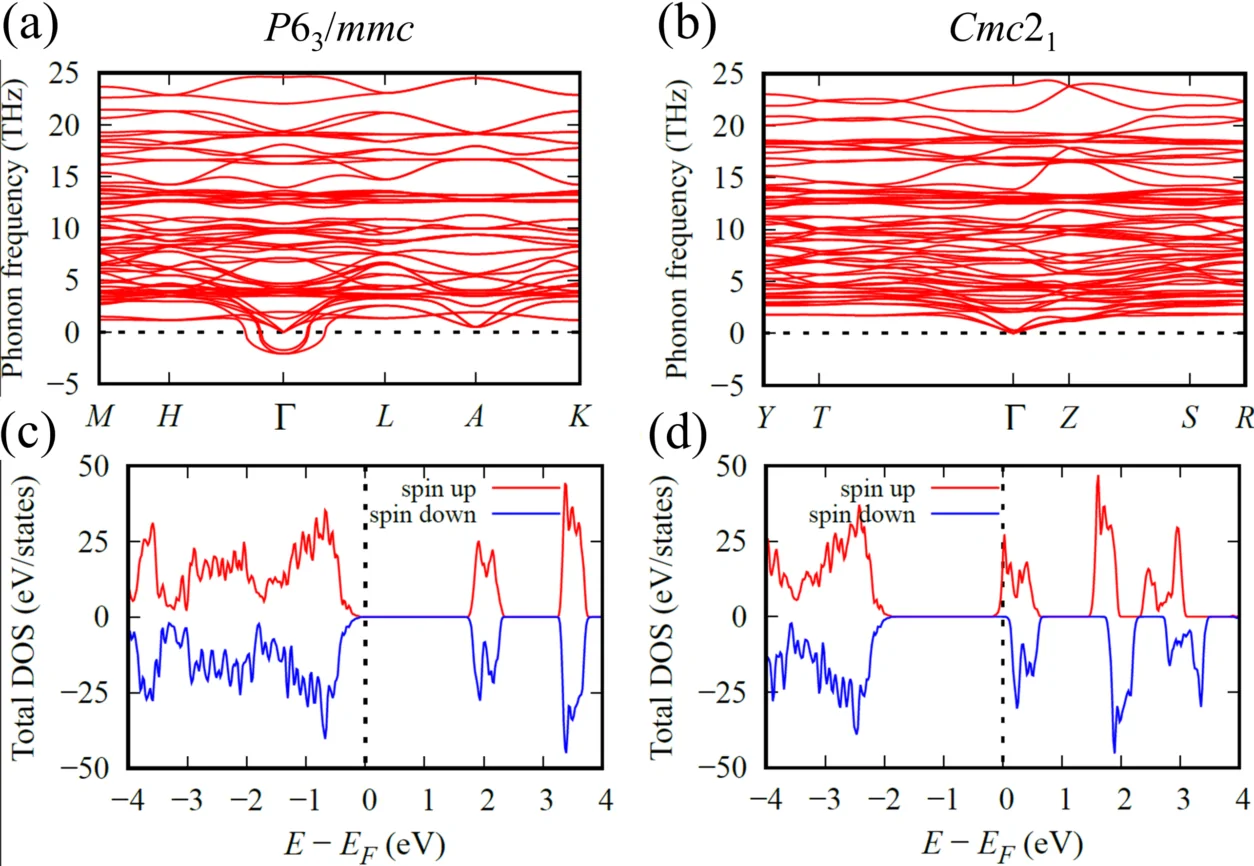
Phonon spectra calculated for (a) the high symmetry *P*6_3_/*mmc* structure and (b) the ground-state *Cmc*2_1_ structure. (c), (d) Total density of states (TDOS) computed for the ground-state *Cmc*2_1_ structure without and with electron doping, respectively. Pristine 4H-SrMnO_3_ is insulating with an electronic band gap of ∼1.73 eV, while a small amount of electron doping (0.1 electrons per Mn) drives the system into a weakly metallic state.

Our structural reinvestigation establishes that the Γ_5_^–^ tilts have the capacity to induce a spontaneous polarization in the 4H polytype. Our attempts to collect dielectric data on our own samples of 4H-SrMnO_3_ were prohibited by large leakage currents (Supporting Note 8). Nevertheless, several signatures of ferroelectricity have been reported recently in 4H-SrMnO_3_ ceramics, including dielectric anomalies near *T*_N_ and *T*_S_ ^54,55^ as well as successful polarization switching.^56^ These signatures were previously attributed to short-range compositional ordering or local structural fluctuations, but our present diffraction insights resolve the origins of these anomalous ferroelectric signatures as being driven by a purely geometric mechanism. This interpretation is supported by our DFT calculations, which reveal that the lowest-frequency mode at the zone center corresponds to the Γ_5_^–^ RUM, and it alone is sufficient to favor the *Cmc*2_1_ phase, even in the absence of any intrinsic Γ_2_^–^ polar instability (Figure 3 and Table S4). The lack of any such instability of the Γ_2_^–^ polar mode demonstrates that the induced polarization is of a purely improper origin. The *Cmc*2_1_ phase is lower in energy than the *C*222_1_ structure by ∼0.092 meV/f.u., which is well above the energy resolution of our DFT calculations (∼0.01 meV/f.u.). The relaxed *Cmc*2_1_ structure is characterized by a small but measurable polarization of ∼0.0245 μC cm^–2^ (Table S4), comparable to that observed in magnetically induced ferroelectrics such as TbMnO_3_.^57^ However, in contrast to such systems where the polarization is contingent on the low-temperature magnetic order, the structural mechanism of the FE order in 4H-SrMnO_3_ enables it to persist well above room temperature. This is due to the intrinsic stiffness associated with face-sharing polyhedra, which favors higher phase transition temperatures,^58^ so inversion-breaking RUMs in other edge- or face-sharing framework structures will likely also exhibit high phase transition temperatures.

While our results demonstrate that distortions transforming as Γ_5_^–^ induce FE order in 4H-SrMnO_3_, this irrep is spanned by a total of six degrees of freedom (Table S2). Because the tilting of the Mn_2_O_9_ bioctahedra involves only one degree of freedom, it is necessary to verify that the observed FE distortion is driven primarily by the RUM itself, rather than by the other degrees of freedom transforming under the same irrep. Such non-RUM degrees of freedom may be characterized by a nontrivial compositional dependence that would frustrate efforts to systematically induce and tune the FE distortion. To this end, we decomposed the refined Γ_5_^–^ distortion in terms of a linear combination of symmetry-adapted displacement modes and compared them against the predicted displacement pattern of the Γ_5_^–^ RUM determined by the ISOTILT webtool (Supporting Note 4). All six symmetry-adapted Γ_5_^–^ displacive modes were allowed to refine freely in our original refinements so as to avoid bias toward the expected RUM displacements. Despite this, we find that ∼99% of the total Γ_5_^–^ distortion can be described by the renormalized RUM displacements alone (Table S8). The residual distortion predominantly stems from minor Sr^2+^ displacements that do not form part of the octahedral framework; thus, this represents the principal non-RUM degree of freedom transforming under the Γ_5_^–^ irrep, which is active in the *Cmc*2_1_ structure. Such *A*-site displacements commonly occur in tilted 3C perovskites and can be generally rationalized as an improper response to the primary framework distortion,^7^ so we conclude that these Sr displacements have a similar physical origin in the 4H polytype as well.

To quantify the distinction between the framework and the *A*-site displacements, we decomposed the total mode amplitude obtained from symmetry-mode Rietveld refinements of variable-temperature I11 data (Supporting Note 5) into the contributions from the Mn_2_O_9_ framework (*Q*_fw_) and the Sr sites (*Q*_Sr_) (Figure 2d). By fitting *Q*_fw_ and *Q*_Sr_ separately to critical expressions of the form *Q* ∼ (*T*_S_ – *T*)^β^, we obtained estimates of *T*_S_ and the critical exponent (β). The extracted transition temperatures are consistent within error (*T*_S_ = 444(5) K for *Q*_fw_ and 447(7) K for *Q*_Sr_), but the critical exponents differ significantly. The framework component yields β = 0.28(4), which is characteristic of a tricritical phase transition and consistent with the octahedral tilting transitions in 3C perovskites such as SrZrO_3_.^59^ In contrast, the Sr contribution is characterized by β = 0.63(7), exceeding the typical range expected for an order parameter (β ≤ 0.5). This behavior confirms that the Sr displacements effectively play an improper role in the phase transition and do not drive the observed FE order. Together, these results demonstrate that the stabilization of the Γ_5_^–^ RUM is responsible for the spontaneous polarization in this system.

Having established the role of the Γ_5_^–^ RUM in inducing the polar distortion, we now show that this same distortion can induce a wFM moment within the predominantly AFM spin texture. Previous studies reported that 4H-SrMnO_3_ adopts an A-type AFM structure below *T*_N_ ≈ 280 K, with the Mn^4+^ spins aligned within the *ab* plane.^52^ Our NPD measurements on GEM at ISIS (Figure 4a) confirm this ordering, which transforms as the mΓ_6_^–^ irrep of the paramagnetic *P*6_3_/*mmc* structure (Figure 1d) and is in good agreement with our first-principles calculations (Supporting Note 3). Unlike the canonical ME perovskite BiFeO_3_,^60^ we detect no magnetic diffuse scattering or satellite reflections down to 1.5 K to suggest that a noncollinear magnetic structure manifests. However, DC magnetic susceptibility measurements (Figure 4b) reveal a clear divergence between zero-field-cooled (ZFC) and field-cooled (FC) responses below *T*_N_, indicative of a wFM component that we are insensitive to in our NPD data. Hysteresis loops recorded in the range of 200–300 K show that, although there is a very weak FM moment within the sample above *T*_N_ (Figure S15a), there is a clear enhancement in the remanent magnetization (*M*_r_) below *T*_N_ (Figure S15b) so that the AFM transition is accompanied by its own wFM component.

**Figure 4:**
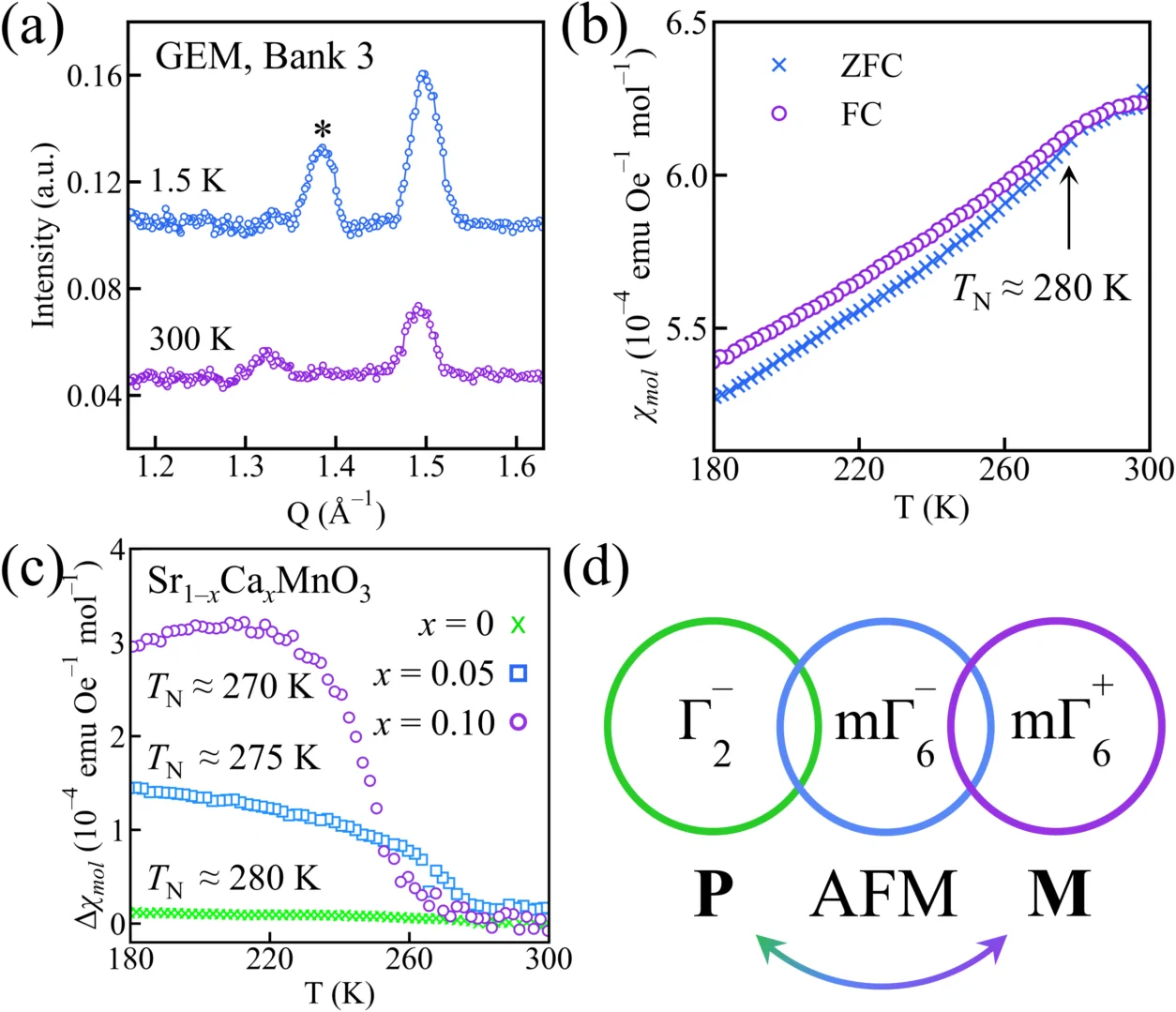
Magnetic properties of 4H-SrMnO_3_. (a) NPD data collected on Bank 3 of GEM, highlighting the emergence of the (002)_mag_ reflection at 1.5 K (*). (b) Molar DC susceptibility (χ_mol_) of 4H-SrMnO_3_, showing the ZFC-FC divergence at *T*_N_. (c) ZFC-FC divergence in χ_mol_ (Δχ_mol_) for the series 4H–Sr_1–*x*_Ca_*x*_MnO_3_, showing the enhancement of the wFM moment with increasing Ca^2+^ substitution. (d) ME coupling in 4H-SrMnO_3_, showing how polarization (**P**) and magnetization (**M**) form a trilinear coupling via the AFM mode.

Symmetry analysis (Supporting Note 6) shows that the observed wFM moment must arise from coupling between the mΓ_6_^–^ AFM order and either the Γ_5_^–^ tilt or the Γ_2_^–^ polar modes, as the mΓ_6_^–^ AFM order by itself is forbidden by symmetry to produce a wFM moment. To verify this, DC susceptibility measurements on a freshly synthesized sample of the untilted 4H-BaMnO_3_ composition (Supporting Note 7) showed no evidence of an equivalent wFM component below *T*_N_, despite it exhibiting the same A-type AFM order in its ground state.^51^ Since *T*_N_ in 4H-SrMnO_3_ is preceded by a regime of short-range AFM ordering,^61,62^ coupling via the tilts and/or the polar distortion likely also explains the residual *M*_r_ we detect above *T*_N_ (Figure S15b).

Symmetry constraints uniquely require the induced wFM moment to transform as the mΓ_6_^+^ irrep (Figure 1e), which is the only mode compatible with the site symmetries of the 4H structure to yield a net ferromagnetic moment. Our noncollinear spin-polarized DFT calculations with SOC confirm that the magnetic easy axis lies along the crystallographic *x*-direction in the *Cmc*2_1_ phase. This permits a wFM mΓ_6_^+^ mode (Figure 1e) yielding Mn moments parallel to those produced by the primary mΓ_6_^–^ AFM order, producing a weak ferrimagnetic (wFiM) ground state described by the Shubnikov group *Cmc*′2_1_′ (Figure 5a). However, although the wFiM state is allowed by symmetry, the insulating nature of 4H-SrMnO_3_ (Figures 3c and S17a) and the absence of charge ordering do not favor a sizable imbalance between the antiparallel Mn Moments, and for this reason, the resulting wFM moment obtained from DFT remains very small (∼0.001 μ_B_). To verify this, we performed charged DFT calculations by adding 0.1 electrons per Mn atom—this drives the system to become weakly metallic, as shown in Figure 3d. Interestingly, even this small level of electron doping induces a finite imbalance between the magnetic moments of the two inequivalent Mn sites within a Mn_2_O_9_ bioctahedral dimer, leading to a nonzero mΓ_6_^+^ wFM mode with an amplitude of ∼0.219 μ_B_. These results suggest that carrier doping, possibly either through introducing oxygen vacancies or aliovalent substitutions on the A and/or B sites, could enhance the symmetry-allowed wFM instability and may provide a promising route toward stabilizing the wFiM state. Our preliminary attempts to prepare reduced SrMnO_3–*x*_ samples confirmed that electron doping leads to a modest enhancement in the wFM moment (Figure S16), demonstrating the validity of this approach.

**Figure 5:**
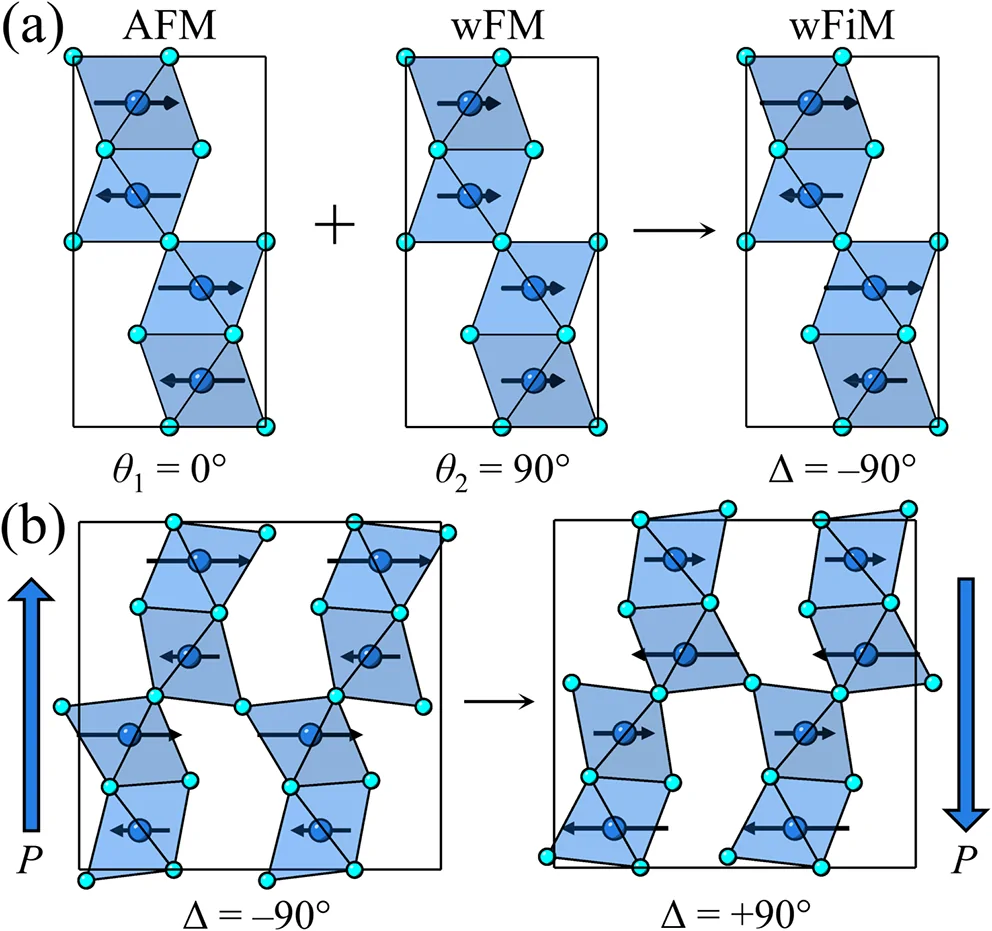
(a) Symmetry-driven generation of a weak ferrimagnetic (wFiM) state via the combination of parallel AFM and wFM moments in the *Cmc*′2_1_′ magnetic structure. (b) A plausible ME switching pathway, showing the redistribution in magnetic moments across the crystallographically inequivalent Mn^4+^ sites. The blue arrows clarify the directions of the net polarization within the structures. The total amplitude of the structural distortion in (b) has been amplified by a factor of 2 for visual clarity.

Although carrier doping can enhance the wFM moment, this will drive the system metallic, so any enhancement will come at the expense of practical FE/ME switching. For this reason, we sought alternative chemical modifications that could enhance the wFM moment without sacrificing the insulating ground state. Because the wFM order is coupled to the Γ_5_^–^ tilts, increasing the tilt magnitude provides a potential route to strengthen the wFM response. To demonstrate the validity of this hypothesis, we found that the partial substitution of 5% and 10% Ca^2+^ (*r* = 1.34 Å) for Sr^2+^ (*r* = 1.44 Å) systematically enhances the wFM component by at least 1 order of magnitude (Figure 4c). This can be rationalized by the smaller ionic radius of Ca^2+^, which increases *A-*site underbonding^50^ and promotes larger tilting of the Mn_2_O_9_ bioctahedra.^46^ Our Rietveld fits against high-resolution S-XRD data on the 5% and 10%-substituted samples corroborate this hypothesis, showing the wFM component increases monotonically with the magnitude of the Mn_2_O_9_ tilts (Supporting Note 9, Figure S21). Importantly, these modest Ca^2+^ substitutions avoid increasing the electronic conductivity while preserving the appealingly high Néel temperature in this system, with *T*_N_ ≈ 270 K for Sr_0.9_Ca_0.1_MnO_3_ (Figure 4c). Clearly, the interdimer Mn–O–Mn superexchange interactions are only weakly perturbed by the isovalent substitution of Ca^2+^ for Sr^2+^, highlighting the strong tuneability of the wFM response through chemical modifications. The improper nature of the induced polarization should also allow compressive strain to enhance both the tilt amplitudes and the polarization/wFM responses, as has been achieved in TbMnO_3_.^63^ This is in contrast to what is typically observed in canonical hybrid-improper ferroelectrics such as Ca_3_Ti_2_O_7_, where a weak proper component due to the Ti^4+^ cations suppresses the polarization upon the application of hydrostatic pressure.^64^

The semiconducting nature of our samples precludes direct observation of ME switching (Supporting Note 8). Indirect ME measurements, such as magnetodielectric measurements, are also impeded by this issue, preventing us from characterizing the precise strength of any ME coupling. However, the symmetry basis of our design strategy allows us to identify the precise nature of the ME coupling (Figure 4d) and propose a plausible ME switching pathway by analyzing the Landau free energy expansion with respect to the high-symmetry paramagnetic phase (Supporting Note 10). The lowest-order coupling between the Γ_2_^–^ polar mode and the AFM/wFM components takes the form

1 $$F∼PM_{1}M_{2}\mathrm{sin\Delta},,\Delta =\theta_{1}-\theta_{2}$$

where *M*_1_ and *M*_2_ are the magnitudes of the symmetrized mΓ_6_^–^ (AFM) and mΓ_6_^+^ (wFM) order parameter components, respectively, and *θ*_1_ and *θ*_2_ are their corresponding phase angles. This closely resembles the form of the ME coupling found in magnetically driven ferroelectrics such as Ni_3_V_2_O_8_,^65^ but we emphasize that the polarization in 4H-SrMnO_3_ is structurally induced (rather than magnetically) via a separate coupling term of the form *F* ∼ *P* *ρ* ^3^ sin 3φ, where ρ and φ are the magnitude and phase of the Γ_5_^–^ order parameter for the Mn_2_O_9_ tilting (Supporting Note 10). For the *Cmc*′2_1_′ ground state determined from our DFT calculations, the relative phase shift between the magnetic order parameter directions is Δ = ± 90°. Physically, this corresponds to an exact parallel alignment between the AFM and wFM moments, resulting in a net wFiM state (Figure 5a). The linear dependence on *P* in eq ([Disp-formula eq1]) permits the system to adopt either a positive or negative polarization to minimize the free energy, so to maintain invariance overall, the transformation *P* → −*P* necessarily switches Δ by ±180° (Figure 5b). Since the switching barrier associated with reorienting the AFM component is likely to be much larger compared to the wFM component, this is most plausibly achieved by reorienting θ_2_ by ±180°, resulting in a redistribution of the magnetic imbalance within the Mn_2_O_9_ bioctahedral dimers. Although the real switching pathway is likely to be far more complex than this (see Supporting Note 10), this coupling nonetheless establishes a feasible basis for the switching of net magnetization under an applied electrical field.

Physically, the crystallographic inequivalence of the Mn^4+^ sites within the bioctahedral dimers is a direct consequence of the improper Γ_2_^–^ polar distortion, which splits the single Mn^4+^ site in the parent *P*6_3_/*mmc* structure into two. This is what permits the imbalance between the Mn^4+^ moments, meaning the induced wFM moment relies on the spontaneous polarization induced within the crystal structure. We confirmed this by performing additional charged DFT calculations for the *C*222_1_ phase—although the system becomes weakly metallic upon electron doping, we found no imbalance between the Mn^4+^ moments to suggest that any wFiM state is stabilized. This suggests that the spin splitting in the doped *Cmc*2_1_ phase is indeed symmetry-driven and does not arise from d-electron correlations. The wFM moment detected in our susceptibility measurements thus reflects the ME coupling scheme we identified from our Landau analysis. Structural signatures of this coupling are inevitably subtle due to the small magnitude of the polarization, but a weak magnetostrictive effect at *T*_N_ corroborates the presence of this coupling. Combined, our experimental and theoretical insights demonstrate the precise mechanistic link between the Γ_5_^–^ tilts, the Γ_2_^–^ polar distortion, and the mΓ_6_^+^ wFM moment (Figure 1f).

From a design perspective, the key advantage of 4H-SrMnO_3_ lies in the simplicity of its ME coupling scheme (Figure 4d). All relevant order parameters transform as zone-centered (Γ-point) irreps of the parent structure, ensuring conservation of crystal momentum and enabling direct coupling between the structural, polar, and magnetic modes. This contrasts with established hexagonal multiferroics such as YMnO_3_,^66,67^ where the coupling involves a zone-boundary K_3_ distortion^68^ and mK_3_ AFM order, formally precluding a net magnetization within the bulk.^69^ Similarly, in conventional 3C perovskites, the high symmetry of the *Pm*3̅*m* aristotype structure requires multiple zone-boundary modes to be condensed simultaneously to achieve comparable coupling.^13^ In contrast, the Γ-point coupling scheme realized here requires only a minimal set of symmetry-breaking distortions—namely, the stabilization of the Γ_5_^–^ tilts (to induce the Γ_2_^–^ polar mode on an improper basis), and A-type AFM order (to allow for a wFM component). Taken together, these results reveal an unusually simple and complete realization of ME coupling within a single symmetry-based framework. To our knowledge, 4H-SrMnO_3_ represents the first experimentally realized system that satisfies such a simple yet robust ME coupling mechanism.^70,71^ Similar behavior has been predicted recently in oxygen-deficient Ruddlesden–Popper phases of the form A_4_B_3_O_9_,^72^ although experimental realization of this remains outstanding. Nevertheless, 4H-SrMnO_3_ provides the essential crystallochemical ingredients required for robust ME coupling, which, coupled with its appealingly high structural and magnetic ordering temperatures, presents promising opportunities for the realization of room-temperature ME switching.

## Conclusions

Our high-resolution diffraction, symmetry-mode analysis, and DFT calculations collectively establish that a single Γ_5_^–^ RUM simultaneously induces a spontaneous polarization and a wFM moment in the 4H polytype. To the best of our knowledge, this is the first example of a single RUM in a crystalline framework capable of breaking global inversion symmetry without the influence of extraneous symmetry-breaking distortions. Similar inversion-breaking RUMs could arise in other framework structures deviating from the conventional perovskite architecture, thus presenting a promising new strategy to design coupled FE and wFM orders near room temperature. The present 4H-*A*MnO_3_ system shows that this can be achieved with minimal symmetry-breaking ingredients, and additional modifications to the *A*/*B*-site size mismatch and/or manipulations of the defect chemistry present further opportunities to enhance the functional properties while preserving the appealingly high ordering temperatures. Although our present work has targeted a ternary ceramic oxide, one of the most appealing facets of our design approach is that it may be readily generalized to other material chemistries. Both halide and hybrid perovskites are of particular interest in this respect due to their propensity to form polytype structures, and the design strategy we outline here can likely be extended to tune the optoelectronic properties, which are of timely interest in these materials. Our work now stimulates the discovery of similar inversion-breaking RUMs in other framework structures that can potentially imbue them with newfound ferroic and/or ME functionalities. Ultimately, this paves new pathways for the rational design of ferroelectric and magnetoelectric materials across more complex chemical architectures than has been possible previously.

## Supplementary Material









## Notes

Landau free energy expansion up to sixth order for the Γ_5_^–^, Γ_2_^–^, mΓ_6_^–^, and mΓ_6_^+^ order parameters (TXT)
Further details on the symmetry analysis, Rietveld refinements, DFT calculations, RUM analysis, magnetic/physical property characterization, and Landau analysis (PDF)
*Cmc*2_1_ structural model refined against synchrotron XRD data collected at 10 K (CIF)
*Cmc*2_1_ structural model refined against synchrotron XRD data collected at 290 K (CIF)
