# Supplementary material for: Near-Room-Temperature Magnetoelectric Coupling Engineered through Inversion-Breaking Tilts in a Bulk Perovskite Polytype
Source: J Am Chem Soc. 2026 Aug 27;148(35):38271–80. doi: 10.1021/jacs.6c11283 (PMC13564441; doi:10.1021/jacs.6c11283)
Supplement: Supplementary file 1 [file ja6c11283_si_002.pdf]

Supplementary Information for: "Near-room-temperature magnetoelectric coupling engineered through inversion-breaking tilts in a bulk perovskite polytype"

Struan Simpson[1], Urmimala Dey[2], Martin R. Lees[3], Ivan Da Silva[4], Nicholas C. Bristowe[5], and Mark S. Senn[1]**

[1] Department of Chemistry, University of Warwick, Gibbet Hill, Coventry, CV4 7AL, U.K.

[2] Luxembourg Institute of Science and Technology, Avenue des Hauts-Fourneaux 5, L4362, Esch-sur-Alzette, Luxembourg

[3] Department of Physics, University of Warwick, Gibbet Hill, Coventry, CV4 7AL, U.K.

[4] ISIS Facility, Rutherford Appleton Laboratory, Harwell Oxford, Didcot OX11 0QX, U.K.

[5] Centre for Materials Physics, Durham University, South Road, Durham, DH1 3LE, U.K.

Supplementary Note 1. Relationship between the Γ_5^- tilt axis and the structural symmetry.

Figure S1 depicts the relationship between the order parameter space of the Γ_5^- tilt mode and the symmetry of the crystal structure. The full basis spanned by the Γ_5^- (a,b) irrep encompasses $C222_1$, $Cmc2_1$, and $P2_1$ symmetries, which correspond to representative order parameter directions (OPDs) of (a,0), (a,$\sqrt{3}$a/3), and (a,b), respectively. The high-symmetry OPDs can be mapped in terms of two sets of sixfold structural domains possessing either $C222_1$ or $Cmc2_1$ symmetry. By writing the OPD in terms of a phase angle $\varphi = \tan^{-1}(b/a)$, these correspond to $\varphi = n\pi/3$ and $(2n+1)\pi/6$, respectively, where n is an integer, hence symmetry-equivalent structural domains are related to each other by $\Delta\varphi = \pm\pi/3$. Physically, the differences between these structures can be understood on the basis of the orientation of the tilt axis within the hexagonal basal plane (Figure S1b and S1c). Due to the face-sharing geometry between pairs of octahedra, the octahedral *B*-site atoms necessarily displace perpendicularly to the tilt axis as part of any proposed rotation (see Fig. 1c in the main text). The displacements are antiparallel across hexagonal close-packed AX_3 layers (i.e., across the face-sharing octahedra), while they are parallel across cubic close-packed AX_3 layers (i.e., across the corner-sharing octahedra).

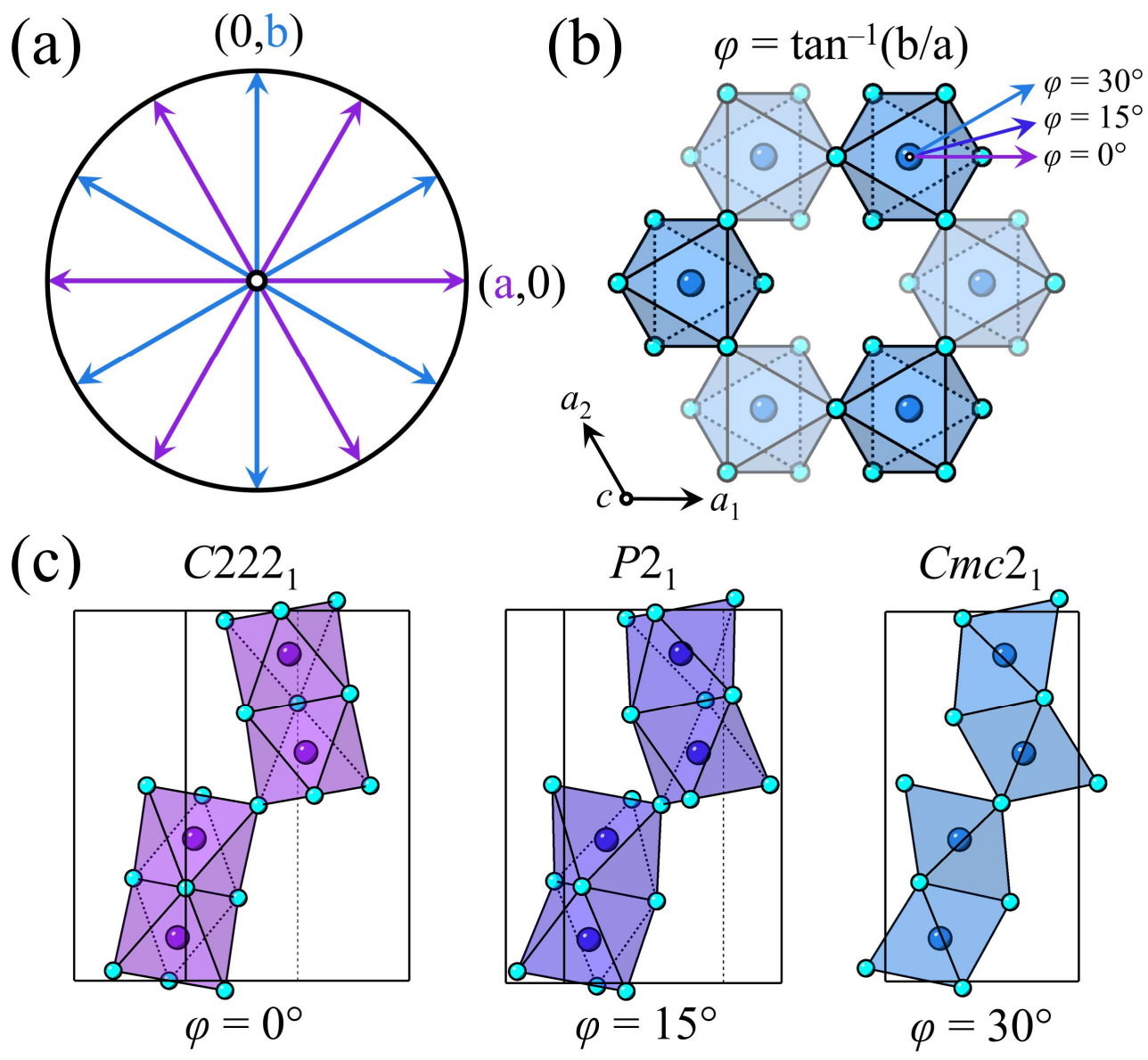

Figure S1. (a) The full order parameter space spanned by the Γ_5^- (a,b) irrep. Symmetry-related domains are depicted using arrows of the same color, with purple and blue representing $C222_1$ and $Cmc2_1$ domains, respectively. (b) Partial map of the Γ_5^- order parameter space projected onto the crystal structure of the 4H polytype viewed down the *c* axis. Three representative tilt axes have been depicted in terms of $\varphi = \tan^{-1}(b/a)$. The unit cell axes are labelled with respect to the hexagonal parent structure. (c) Views of the crystal structure along the three tilt axes shown in (b). The parent hexagonal cell has been superimposed on each, and the *A*-site atoms have been omitted for visual clarity.

Supplementary Note 2. Determining the ground-state crystal structure of 4H-$SrMnO_3$.

Previous diffraction experiments concluded that the ground-state structure of 4H-$SrMnO_3$ was best described by $C222_1$ symmetry [1]. This prior space group assignment was made on the basis of the appearance of $hh(2\bar{h})l$: $l = 2n + 1$ reflections in selected-area electron diffraction patterns, combined with systematic tests of different structural models against neutron and synchrotron diffraction data based on higher-order isotropy subgroups of the parent $P6_3/mmc$ space group. Based on the $C222_1$ assignment that was made, it was concluded that the low-symmetry structure could be described in terms of its transformation as the Γ_5^- (a,0) irrep of the $P6_3/mmc$ structure. Our own S-XRD experiments on the ID22 beamline showed that this $C222_1$ structural model gave a broadly reasonable description of the observed data (Figure S2), but it fit poorly against the superstructure reflections (Figure S3). We thus sought to re-examine the structural assignment below T_s to deduce whether an alternative choice of space group was more appropriate.

The $C222_1$ and $Cmc2_1$ space groups are both C-centered, hence they are distinguished by the general reflection condition hkl: $h + k = 2n$ (where n is an integer). However, the $Cmc2_1$ space group is distinguished from $C222_1$ by the additional condition $h0l$: $h,l = 2n$, hence an additional set of superstructure reflections is predicted for the $C222_1$ structure compared to the $Cmc2_1$ structure. This provides a systematic basis from which to deduce the correct space group assignment. In general, this leads to the $C222_1$ and $P2_1$ models predicting a splitting of the superstructure reflections, while no such splitting is predicted for the $Cmc2_1$ model (Figure S3). We do not find convincing evidence of such splitting, nor any anomalous broadening with respect to the parent Bragg reflections, within the exceptionally high resolution of our S-XRD experiments ($\Delta d/d \approx 1 \times 10^{-4}$). To verify this, we tested all possible space group assignments spanned by the Γ_5^- (a,b) basis (Figure S1) against our S-XRD data.

Our structural models were constructed as follows. The instrumental contribution to the peak shape was modelled by a simple convolution of fixed Gaussian and Lorentzian contributions, each obtained by refinements against data collected on a Si standard. 21 background terms were refined, including 12 terms of a shifted Chebyschev polynomial and 9 additional parameters describing three Gaussian peaks to account for background contributions from the cryostat. Both a zero-point error and a scale factor were refined in addition to the lattice parameters (3 for the $C222_1$ and $Cmc2_1$ models and 4 for the $P2_1$ model). The internal atomic coordinates were refined freely for each model, with 10, 18, and 30 independent degrees of freedom for the $C222_1$, $Cmc2_1$, and $P2_1$ structures, respectively. Isotropic displacement parameters were refined for each atomic site and constrained to be equal for each element; the only exception was the Sr(1) site, where anisotropic displacement parameters consistently improved the quality of fit (~2% reduction in R_{wp}) for each model. Overall, this resulted in 7 additional refined parameters for the $C222_1$ and $Cmc2_1$ models and 9 for the $P2_1$ model. Finally, we also included 2 crystallite size broadening terms as well as Stephens anisotropic broadening terms (7 parameters for

$C222_1$ and $Cmc2_1$; 10 for $P2_1$) to model sample-dependent contributions to the peak shapes. This resulted in a total of 52, 60, and 78 refined parameters for the $C222_1$, $Cmc2_1$, and $P2_1$ models, respectively. These were then fitted against the data collected at 10 K, 100 K, 200 K, and 290 K, thus spanning a range of temperatures below T_s to ensure sensitivity to the correct space group assignment.

The fitting statistics obtained from each model are summarized in Table S1. At 290 K and 200 K, we find that little distinguishes the three possible choices of structural model, potentially explaining prior ambiguity regarding the true space assignment below T_s. However, at 100 K and 10 K, we find that the differences between the three structural models become more pronounced. This directly reflects the enhanced ferroelastic (i.e., orthorhombic) strain at low temperatures, hence powder diffraction provides us with sufficient sensitivity to distinguish between the different structural models. Based on the combination of fit quality and the number of fitting parameters, our Rietveld fits show that the $Cmc2_1$ model provides the best description of the low-temperature data. This is further supported by its ability to describe the low-temperature superstructure reflections more accurately than the canonical $C222_1$ model (Figure S3). The $P2_1$ model is distinguished from the $Cmc2_1$ model by an additional 18 refined parameters, so the modest improvement for this model can be attributed to overparameterization.

Table S1. Summary of fitting statistics for Rietveld-fits performed against synchrotron XRD (S-XRD) data at select temperatures below the structural phase transition. The $C222_1$, $Cmc2_1$, and $P2_1$ models had 52, 60, and 78 refined parameters, respectively.

T (K)	Model	S-XRD R_{wp} (%)
290	$C222_1$	7.29
	$Cmc2_1$	7.27
	$P2_1$	7.11
200	$C222_1$	7.73
	$Cmc2_1$	7.65
	$P2_1$	7.29
100	$C222_1$	8.38
	$Cmc2_1$	7.79
	$P2_1$	7.38
10	$C222_1$	9.28
	$Cmc2_1$	8.15
	$P2_1$	7.40

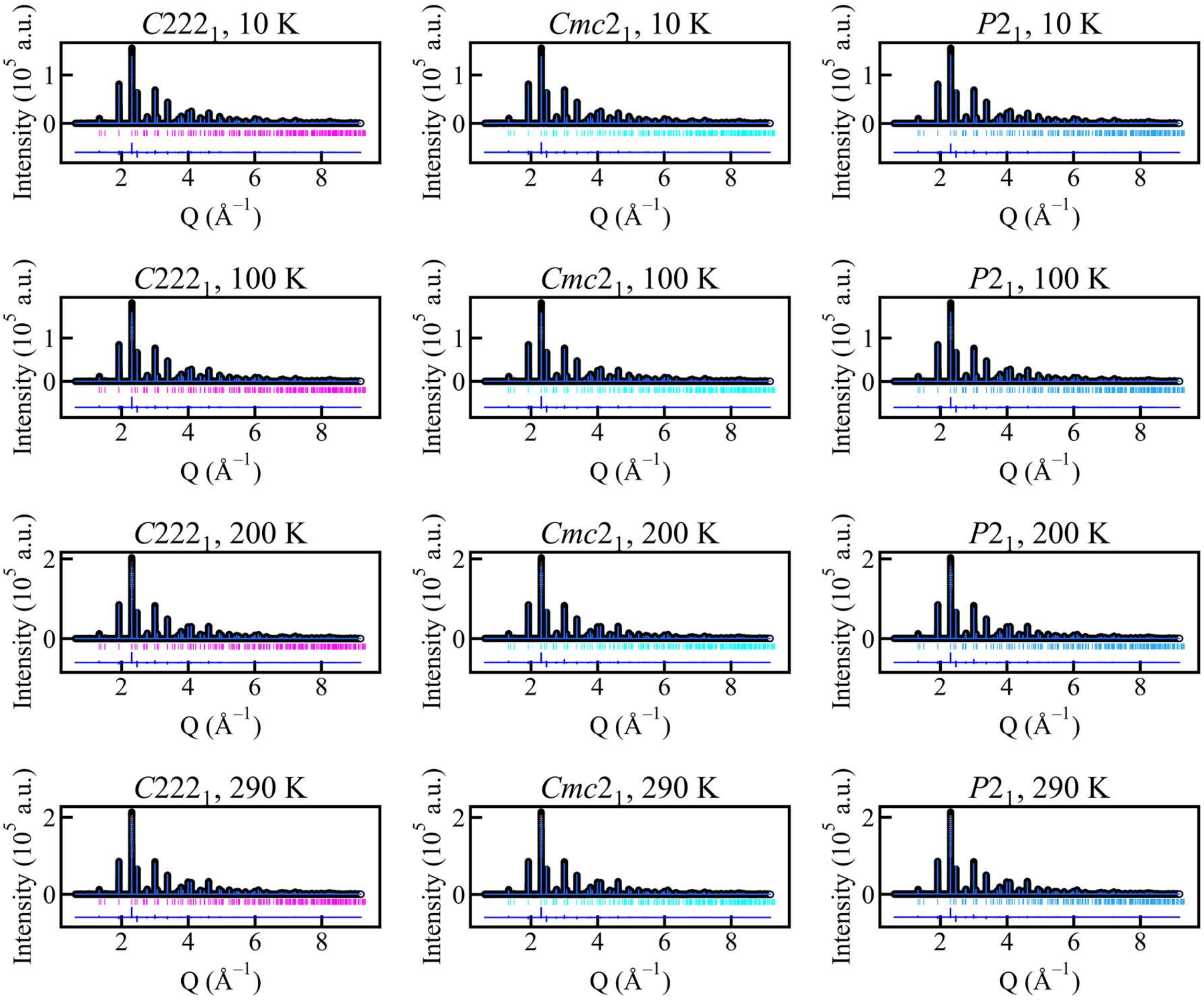

Figure S2. Rietveld fits of the $C222_1$, $Cmc2_1$, and $P2_1$ structural models against S-XRD data collected on ID22 at select temperatures below T_s for 4H-$SrMnO_3$. Pink, cyan, and blue tick marks represent the positions of the expected Bragg reflections for the $C222_1$, $Cmc2_1$, and $P2_1$ models, respectively.

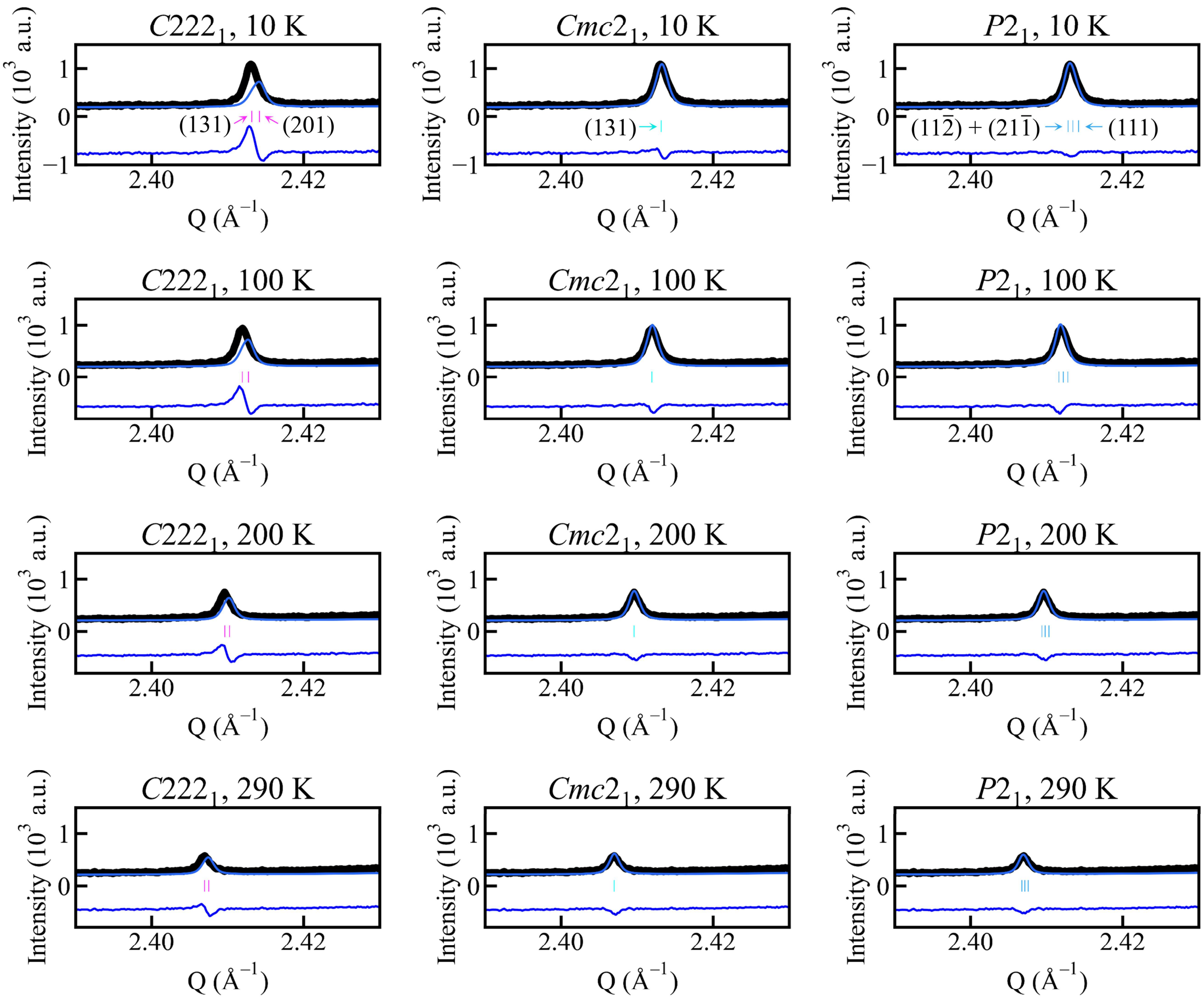

Figure S3. Expanded view of the Rietveld fits against the ID22 data for 4H-$SrMnO_3$, highlighting the poor fit of the *C*222$_1$ structural model and the good fits of the *Cmc*2$_1$ and *P*2$_1$ models against the first observed superstructure reflection. The predicted reflections for each model have been highlighted and indexed with respect to their corresponding space groups in the 10 K pattern. The *C*222$_1$ and *P*2$_1$ structures both predict a subtle splitting of the superstructure reflections, while the *Cmc*2$_1$ model predicts only a single superstructure reflection. At 10 K, the ultra-high resolution of our S-XRD data ($\Delta d/d \approx 1 \times 10^{-4}$) is sufficient to allow us to distinguish any splitting or broadening of the superstructure reflections, but as shown by the fits against the *C*222$_1$ and *P*2$_1$ model, this is either not consistent with the observed pattern or unnecessary to model the observed superstructure reflections. Any departure from the orthorhombic metric symmetry must therefore comprise of a strain of less than 10^{-4}.

Normally, the challenge of distinguishing the precise orientation of the tilt axis with respect to the crystal structure would be intractable by Rietveld refinement alone as the tilts predominantly involve displacements of the oxygen atoms (Table S2). This means the *C*222$_1$, *Cmc*2$_1$, and *P*2$_1$ models will be mostly distinguished by subtle intensity differences in the calculated patterns, and the peak intensities will be strongly correlated to the other refined parameters in our model. However, the enhanced sensitivity of S-XRD to precise peak positions allows us to exploit the improper ferroelastic strain

transforming as the two-dimensional Γ_5^+ (c,d) irrep to infer the Γ_5^- tilt OPD; this is precisely why we make our structural assignment on the basis of our S-XRD data, rather than the NPD data (despite the greater sensitivity of the latter to the precise oxygen positions). The $C222_1$ and $Cmc2_1$ structures may be mapped to distinct Γ_5^+ OPDs ((a,$-\sqrt{3}$a) and (a,0), respectively). To encode this in our refinements, we parameterized the Γ_5^+ strain as an order parameter of the form (ε, θ), where $\varepsilon = (c^2 + d^2)^{1/2}$ and $\theta = \tan^{-1}(d/c)$. Much like the Γ_5^- tilt mode, this allows us to write the Γ_5^+ strain OPD in terms of a phase angle θ and construct a series of structural models which linearly interpolate between the $C222_1$ and $Cmc2_1$ OPDs in terms of θ. A trivial mapping between the bases of the Γ_5^+ and Γ_5^- irreps then allows us to rewrite θ in terms of the Γ_5^- OPD (i.e., φ) as $\theta = 2\varphi - \pi/3$. Thus, although our models are strictly speaking parameterized with respect to the Γ_5^+ strain, they can be directly interpreted in terms of the Γ_5^- tilt OPD. Note that, due to the polycrystalline nature of our samples, we are insensitive to the absolute orientation of the Γ_5^+ strain OPD. Nevertheless, we retain sensitivity to its relative orientation so that we can still distinguish the average crystallographic symmetry across the bulk with this approach.

To perform our significance tests, our refinements were constructed as follows. The internal atomic degrees of freedom were fixed based on the displacive mode components obtained from a $Cmc2_1$ symmetry-mode refinement performed against the 10 K dataset. Since changes to these displacement modes only effect minor intensity differences to the calculated patterns, this constraint minimizes the number of refined parameters and helps to ensure any significant changes in fitting statistics can be attributed to the systematic variation in θ (hence, φ) across the different structural models. We also chose to perform our refinements using isotropic displacement parameters that were constrained based on their refined values at 10 K. We then refined a global scaling parameter for these displacement parameters for the higher-temperature datasets. The anisotropic Stephens terms were also replaced with simple isotropic strain broadening terms to minimize the total number of refined parameters in our model. Ultimately, the only freely refined parameters in our significance tests were the background terms, the zero-shift and scale parameters, the Γ_1^+ strain modes (to account for thermal expansion), and $\rho(\Gamma_5^+)$. Additionally, each structural model corresponding to different choices of φ was refined on an individual basis (rather than a sequential basis) so as to avoid introducing bias into the refinements.

Fig. 2c shows the results of our significance tests at select temperatures below 290 K. At 10 K, we find that the refinement surface is characterized by a broad minimum around the $Cmc2_1$ OPD ($\varphi = 30°$), while the $C222_1$ OPD ($\varphi = 60°$) represents a clear maximum. This is also true for the data collected at 100 K, suggesting $Cmc2_1$ symmetry persists up to at least this temperature. The differences between the two high-symmetry OPDs are much smaller at higher temperatures. This is due to the reduction in magnitude of the ferroelastic strain (ε at 200 K is ~20% of the 10 K value), so we are less sensitive to the precise space group assignment at higher temperatures. Nevertheless, the appearance of the superstructure reflections remains broadly unchanged up to 290 K (Figure S3), and we find no evidence

of anomalous broadening compared to the parent Bragg reflections at higher temperatures to suggest there is any additional phase transition away from the polar structure within the very high resolution of our S-XRD experiments ($\Delta d/d \approx 1 \times 10^{-4}$). Therefore, our significance tests demonstrate that the $Cmc2_1$ model is the most appropriate structural assignment for the tilted structure of 4H-$SrMnO_3$.

The final fitting parameters obtained from our $Cmc2_1$ structural model are shown in Figure S4. The transformation matrix relating the parent lattice vectors of the $P6_3/mmc$ structure to the $Cmc2_1$ basis is:

$$\begin{pmatrix} 1 & 0 & 0 & 0 \\ 1 & 2 & 0 & 0 \\ 0 & 0 & 1 & 0 \end{pmatrix}.$$

We find no evidence of any volume discontinuity across the full temperature range spanned by our S-XRD experiments, substantiating that no additional phase transitions occur up to T_s. This is in contrast to the 6H polytype of $BaTiO_3$, where its $Cmc2_1 \rightarrow C222_1$ transition is marked by a clear ferroelastic anomaly as well as an exotic Goldstone regime which is readily distinguished by significant diffuse-like scattering between twin-related diffraction peaks [2]. The amplitudes of the refined Γ_5^- tilt mode components at 10 K are provided in Table S2, and the Mn–O bond lengths are provided for reference in Table S3.

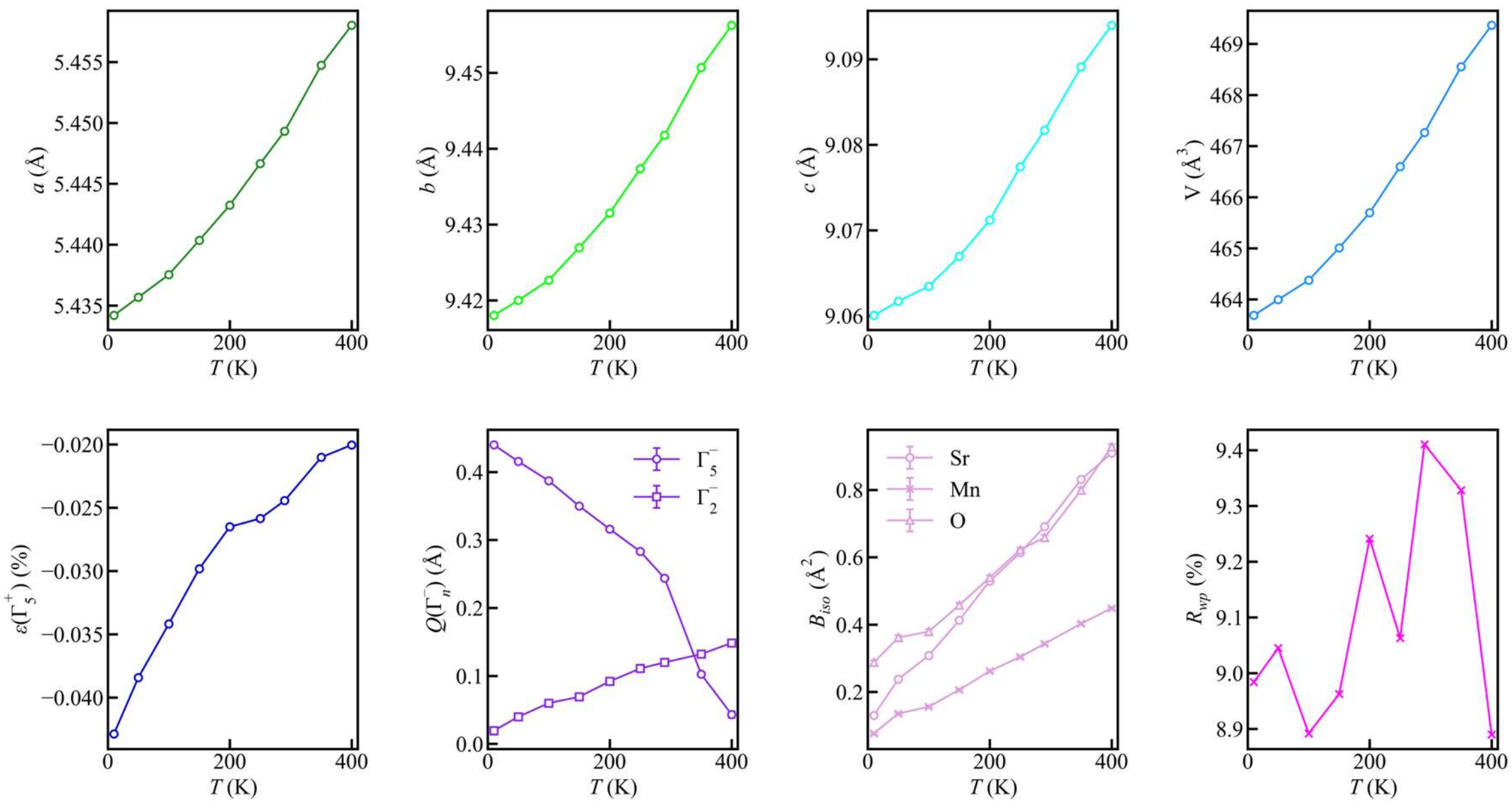

Figure S4. Selected fitting parameters from the final $Cmc2_1$ structural model refined against S-XRD data collected on ID22 (ESRF, France) for 4H-$SrMnO_3$ in the range 10–400 K. Where not apparent, the error bars are smaller than the data points. For these refinements, only isotropic displacement parameters were refined to minimize the total number of refined parameters. The total Γ_5^- tilt and Γ_2^- polar mode amplitudes (Q) were calculated based on the unconstrained mode components (q_i) as $Q = (\Sigma q_i^2)^{1/2}$. Note the apparent increase in $Q(\Gamma_2^-)$ at higher T is an artefact of the refinements due to the use of isotropic displacement parameters.

Table S2. The Γ_5^- tilt mode components from the $Cmc2_1$ structural model refined against the S-XRD data collected at 10 K for 4H-$SrMnO_3$. The relative weights of each component have been expressed as a percentage of the total mode amplitude, and the maximum displacement on each site (d_{max}) has been shown for reference. The O(1) and O(2) sites – corresponding to the oxygen atoms at the corner-sharing and face-sharing positions, respectively – form the dominant contribution to the total mode amplitude.

Site	Symmetry	$Q(\Gamma_5^-)$ (Å)	Weight (%)	d_{max} (Å)
Sr(1)	E_u	0.09653	5.5	0.06826
Mn	E	0.12837	9.8	0.06418
O(1)	A_u	0.22994	31.3	0.11497
	B_{1u}	–0.24076	34.3	0.13900
	B_{2u}	0.13036	10.1	0.07526
O(2)	B_1	–0.12300	9.0	0.07101
Total		0.41080	100.0	

Table S3. Selected bond lengths obtained from Rietveld fits against the 10 K S-XRD data using the $Cmc2_1$ structural model.

Sites	Distance (Å)	Sites	Distance (Å)
Mn(1)–O(1)	1.852(2) x 2	Mn(2)–O(1)	1.882(2) x 1
	1.871(2) x 1		1.899(2) x 2
Mn(1)–O(2)	1.863(2) x 2	Mn(2)–O(2)	1.904(3) x 1
	1.875(3) x 1		1.946(2) x 2
<Mn(1)–O>	1.86(1) x 6	<Mn(2)–O>	1.91(2) x 6
Mn(1)–Mn(2)	2.4866(2) x 1		

Supplementary Note 3. DFT calculations.

Phonon calculations for the aristotype $P6_3/mmc$ structure reveal that the lowest frequency phonon mode at the zone center transforms as the Γ_5^- irrep (Fig. 3a). Condensation of the doubly degenerate Γ_5^- tilt instability along different order parameter directions results in a piezoelectric $C222_1$ phase and two polar phases with $Cmc2_1$ and $P2_1$ symmetries. The next unstable phonon mode transforms as the Γ_3^- irrep and corresponds to another piezoelectric phase with $P\overline{6}2c$ symmetry. This is in perfect agreement with our previous RUM analysis, which showed that the Γ_3^- tilt mode represents the only other Γ-point RUM in the 4H polytype [3]. No intrinsic Γ_2^- polar instability is observed in the phonon spectrum, and it solely appears as a secondary order parameter in the $Cmc2_1$ and $P2_1$ structures when the Γ_5^- tilt mode is condensed. Total energy calculations (Table S4) further confirm that the $Cmc2_1$ phase has the lowest energy, in agreement with experiment, and exhibits a dynamically stable phonon dispersion (Figure 3b).

Our DFT data demonstrate that the ground-state $Cmc2_1$ structure is characterized by a non-zero polarization, with a tilt amplitude of $Q(\Gamma_5^-) \approx 0.81$ Å and a polar mode amplitude of $Q(\Gamma_2^-) \approx 0.04$ Å. Under the point-charge approximation, the polar amplitude corresponds to a ground-state polarization of $P \approx 0.12$ μC cm^{-2}, which is comparable to the magnitude of spontaneous polarization found in other bulk improper ferroelectrics such as $TbMnO_3$ [4]. Polarization calculation using the Berry phase method results in a smaller value of ≈ 0.0245 μC cm^{-2}. Nevertheless, as first-principles calculations tend to mismatch the observed magnitude of the polarization in improper ferroelectrics, we used the Γ_2^- polar mode components from the DFT-relaxed structure to estimate the experimental polarization based on our high-resolution S-XRD data. It can be extremely challenging to model polar distortions in improper ferroelectrics by Rietveld refinement alone as the weak nature of the polar distortions commonly effects only minor intensity differences to the existing Bragg reflections of the parent structure, hence polar mode amplitudes tend to be strongly correlated to other refined parameters. In the case of 4H-$SrMnO_3$, the freely refined polar mode amplitudes consistently increased with increasing temperature (Figure S4), reflecting their strong correlation to the (anisotropic) displacement parameters on the Sr(1) site. For this reason, we can only reliably model the polar distortion at 10 K where correlations between the refined polar mode amplitude and the displacement parameters will be minimal. By constraining our structural model in terms of the individual mode components extracted from the relaxed DFT structure, we obtained $Q(\Gamma_5^-) \approx 0.442(1)$ Å and $Q(\Gamma_2^-) \approx 0.018(8)$ Å. Both are approximately 50% of the DFT-relaxed amplitudes, showing relatively good agreement between the experimental and theoretical mode amplitudes.

To determine the magnetic ground state from DFT, we consider three different spin configurations: ferromagnetic (FM), A-type antiferromagnetic (A-AFM), and collinear ↑↑↓↓ AFM orderings, as shown in Figure S5. Our calculations confirm that the A-AFM ordering has the lowest energy, lying 54.737

meV/f.u. and 3.560 meV/f.u. below the FM and ↑↑↓↓ AFM orderings, respectively, in agreement with experimental observations and previous DFT studies [5].

As discussed in the main text, the wFM moment obtained for 4H-$SrMnO_3$ remains very small due to its insulating nature and the absence of any charge ordering on the Mn sites. To enhance the wFM instability and provide a microscopic route toward stabilizing the wFiM state, we performed charged DFT calculations by adding 0.1 electrons per Mn atom, resulting in a finite imbalance between the magnetic moments of the two inequivalent Mn sites, as shown in Table S6.

Table S4. Relative energies and mode details of DFT-relaxed structures of 4H-$SrMnO_3$. Relative energies (ΔE) have been reported relative to the lowest-energy structure.

Phase	$P6_3/mmc$	$P6_3mc$	$C222_1$	$Cmc2_1$	$P2_1$
ΔE (meV/f.u.)	14.802	13.140	0.092	0.000	0.157
Primary irrep (Å)	N/A	Γ_3^- (a): 0.35376	Γ_5^-(a,0): 0.81656	Γ_5^-(a,$\sqrt{3}$a/3): 0.81652	Γ_5^-(a,b): 0.81746
Secondary irreps (Å)	N/A	Γ_1^+(a): 0.00985	Γ_1^+(a): 0.02213<br>Γ_5^+(a,$\sqrt{3}$a): 0.07289<br>Γ_1^-(a): 0.00998	Γ_1^+(a): 0.02215<br>Γ_5^+(a,0): 0.06397<br>Γ_2^-(a): 0.04041	Γ_1^+(a): 0.02231<br>Γ_2^+(a): 0.00004<br>Γ_5^+(a,b): 0.07180<br>Γ_1^-(a): 0.00888<br>Γ_2^-(a): 0.01321
Strain modes	N/A	N/A	Γ_5^+(a,$\sqrt{3}$a): -0.00130	Γ_5^+(a,0): -0.00163	Γ_5^+(a,b):<br>a = -0.00027<br>b = 0.00133

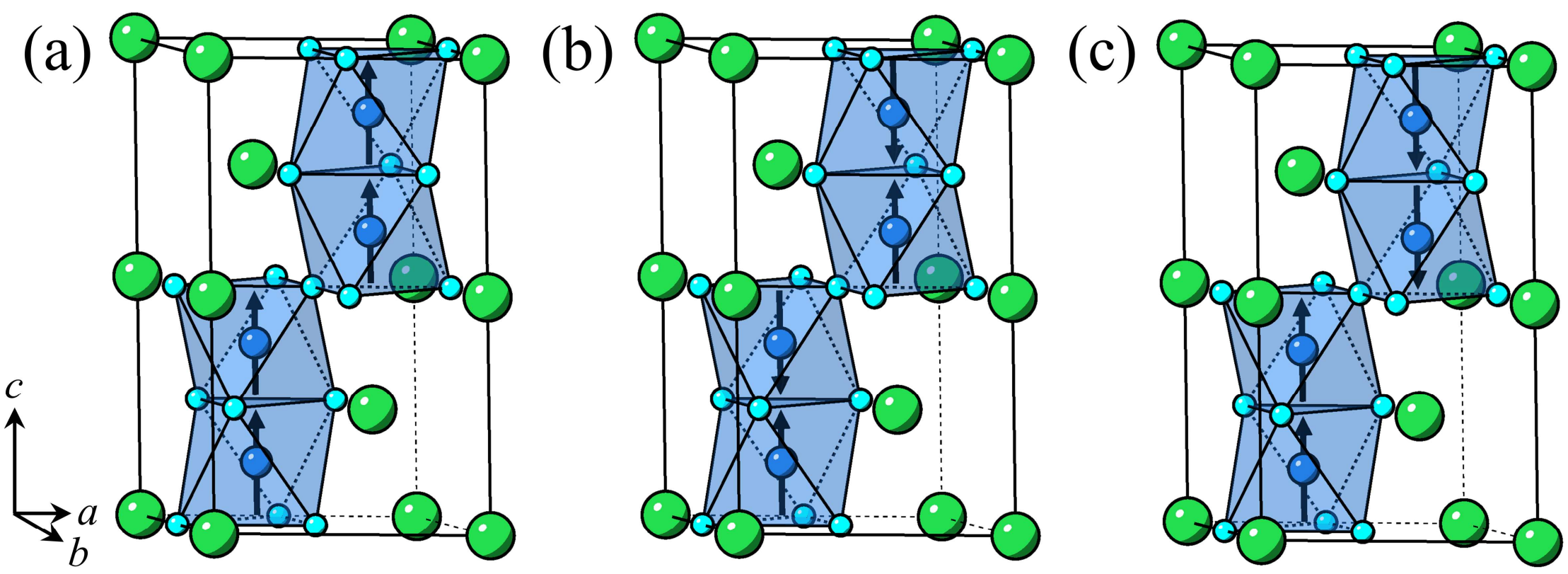

Figure S5. Collinear magnetic configurations considered in DFT calculations to determine the magnetic ground state: (a) Ferromagnetic (FM), (b) A-type antiferromagnetic (A-AFM), and (c) collinear ↑↑↓↓ AFM orderings. Green, blue, and cyan spheres correspond to Sr, Mn, and O atoms, respectively. Black arrows indicate the directions of Mn spin moments.

Table S5. Relative energies of the A-AFM ground state for different spin-axis orientations obtained from non-collinear DFT calculations including SOC with U_{eff} = 4.5 eV applied to the Mn 3d orbitals. U_{eff} was varied within a reasonable range to verify the stability of the magnetic easy axis direction.

Spin axis direction	*x*	*y*	*z*
ΔE (meV/f.u.)	0.000	0.007	0.073

Table S6. Magnetic mode details for the undoped and electron-doped phases in the A-AFM ground state with the magnetic easy-axis orientation. In the pristine phase, the two inequivalent Mn sites possess antiparallel magnetic moments with nearly identical magnitudes of 2.983 μ_B and 2.982 μ_B, yielding a very small wFM moment. On the other hand, electron doping by 0.1 electrons per Mn induces a finite imbalance between the magnitudes of the antiparallel Mn moments, with values of 3.156 μ_B and 2.937 μ_B, resulting in a finite amplitude of the wFM mode.

Phase	Undoped (insulating)	Electron doped (weakly metallic)
Primary magnetic irrep (μ_B)	$m\Gamma_6^-$ (a,0): 5.97043	$m\Gamma_6^-$ (a,0): 6.09855
Secondary magnetic irrep (μ_B)	$m\Gamma_6^+$ (0,a): 0.00100	$m\Gamma_6^+$ (0,a): 0.21920

Supplementary Note 4. RUM analysis of the Γ_5^- distortion.

Our present work is informed by our prior enumeration of the rigid-unit modes (RUMs) in various polytypes of the perovskite structure [3] using the ISOTILT webtool [6]. This tool can also be used to generate the list of atomic positions which are produced by each RUM, and the corresponding distorted structure can be decomposed in terms of symmetry-adapted displacement modes of the parent structure and compared against an observed structural distortion to determine whether it is consistent with the predicted displacement pattern of a given RUM. This allows RUM-based contributions to be distinguished from extraneous degrees of freedom (e.g., cation/anion displacements, Jahn-Teller distortions, strain) which transform under the basis of the same irrep. Normally, this information is trivial to discern in simple structure types such as 3C perovskites whose RUMs can be readily orthogonalized from extraneous distortions by virtue of a symmetry-adapted basis alone. However, for more complex structures, RUMs may transform as a linear combination of several distinct displacement modes acting on distinct Wyckoff sites which nonetheless collectively transform under the same irrep, so this irrep encompasses more degrees of freedom than are strictly necessary to reproduce a RUM-type displacement pattern. For these reasons, we sought to develop a simple workflow which could discriminate the RUM contribution to an observed structural distortion.

First, we provide some brief context to the RUM analysis performed by ISOTILT. In summary, ISOTILT constructs a series of symmetry-mode blocks which encompass all of the symmetry-allowed rotational modes of a given superstructure in terms of their irrep transformations with respect to the parent structure. These blocks contain information about how the pivot and shared atoms comprising a given framework transform under each irrep. A singular-value decomposition is then performed on this system of equations, allowing its null space to be analyzed to pinpoint the rotational modes which avoid internal distortions of the rigid units within the parent structure. This yields a series of right-singular vectors (RSVs) whose components delineate linear combinations of symmetry modes encompassing the displacement pattern of a potential RUM; the actual RUM character of each RSV can be assessed by calculating a relative root-mean-squared deviation (RrmsD) of the RSV components. A non-zero RrmsD characterizes an RSV with non-RUM character, while a zero (or negligible) RrmsD characterizes a pure (or quasi-) RUM. For reference, we provide example outputs for the Γ_5^- symmetry-mode blocks in the 4H and 6H polytypes in Table S7, and we show representative structures of the RSVs with the lowest RrmsDs for the two polytypes in Figure S6.

Next, we outline our overall workflow to decompose a given structural distortion in terms of the RSVs identified by ISOTILT. ISOTILT can generate the list of atomic positions produced by the coefficients of a given RSV. These positions can then be used to construct a cif corresponding to a representative structure for each RSV. Using ISODISTORT, the RSV cifs can then be decomposed in terms of the displacive components of the pivot and passenger atoms (e.g., the atoms occupying the *B* and *X* sites,

respectively, in an ABX_3 perovskite). The mode details for each RSV can then be exported, providing a basis onto which the observed structural distortion can be projected. For this, we express the observed distortion $\mathbf{Q}_{obs}$ in terms of a linear combination of RSVs:

$$\mathbf{Q}_{obs} = \mathbf{Q}_0 + \sum_n c_n \mathbf{R}_n \quad (1)$$

where $\mathbf{R}_n$ are the RSVs, c_n are coefficients representing the weight of each RUM, and $\mathbf{Q}_0$ represents the residual structural distortion transforming under the same irrep but not spanned by the RSV basis. The coefficients c_n can be obtained through a least-squares fitting of Eq. (1) against a given $\mathbf{Q}_{obs}$ to decompose the contribution from each RSV. Although not all RSVs necessarily correspond to physically plausible RUM distortions, including the full set of RSVs in the fitting routine provides an unbiased method to determine which RSVs best reproduce the observed distortion. This means we make no prior assumptions based on the RUM character of a given RSV to characterize the observed distortion.

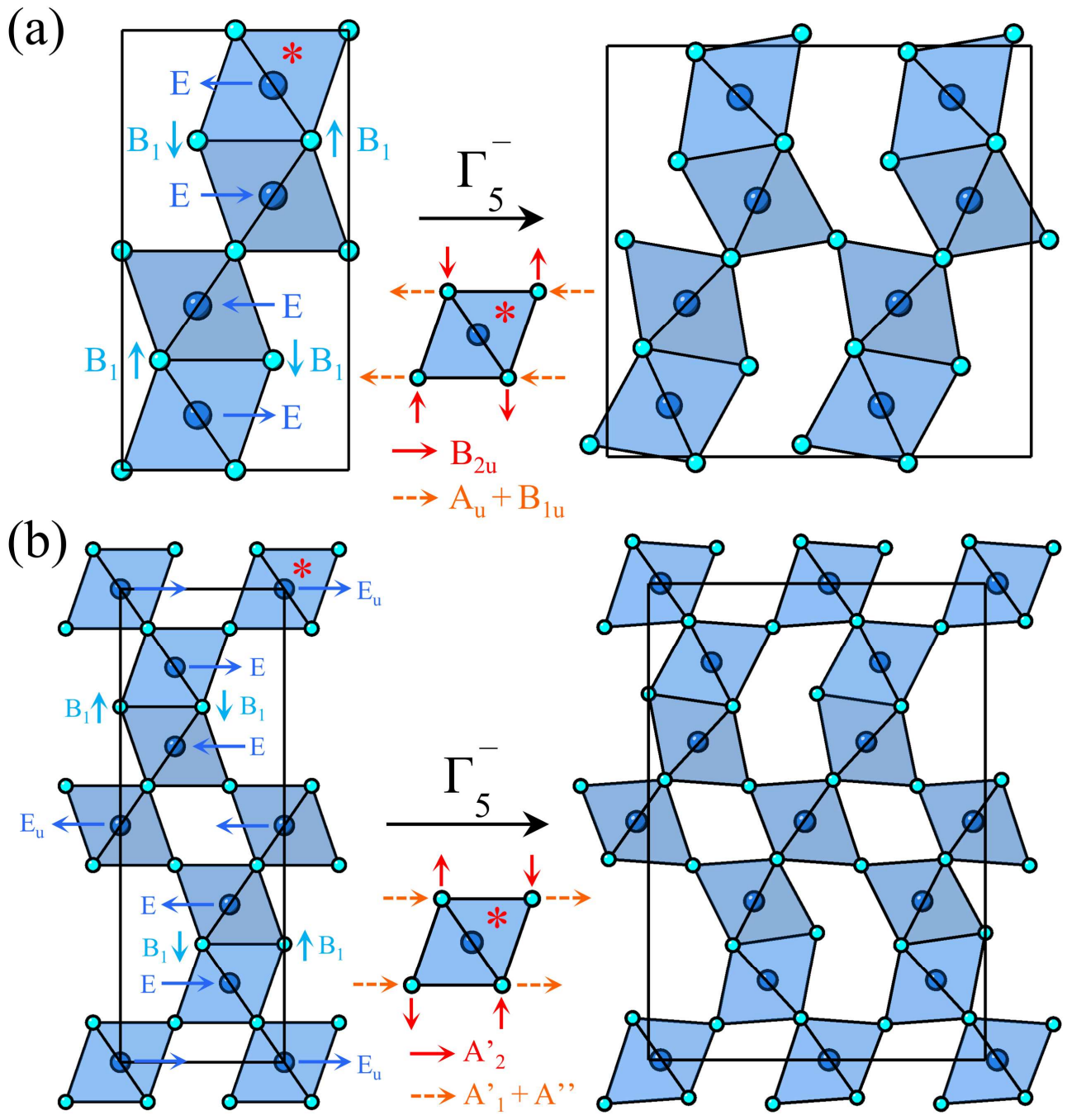

Figure S6. Displacement modes for the Γ_5^- RSVs in the 4H (a) and 6H (b) polytypes. The irreps for the local Wyckoff positions have been labelled, and the corresponding point groups are provided in Tables S8 and S9. The vectors for the corner-sharing O(1) sites have been shown separately for visual clarity, with red asterisks denoting which octahedra the vectors have been depicted with respect to. Representative structures for each transformation were generated based on the RSVs output by ISOTILT; only the RSVs with the lowest RrmsD have been shown for simplicity.

To illustrate our approach, we first consider the experimental mode amplitudes we extracted from our S-XRD refinements for 4H-$SrMnO_3$ (Table S2). Table S8 summarizes the results of our RSV fitting against these mode amplitudes. Each RSV was renormalized based on the total mode amplitude of the observed distortion calculated without the displacement contribution from the Sr atoms – this is because

the Sr atoms are not part of the polyhedral framework, hence they do not form part of any RUM-type motion. We find that the first RSV in Table S7 ($\mathbf{R}_1$) forms the main contribution to the observed amplitudes ($c_1 \approx 1.04$), while the contribution from $\mathbf{R}_2$ is significantly smaller ($c_2 \approx 0.09$). The residual structural distortion is almost entirely weighted towards the non-RUM Sr displacements (which are ~92% of the total residual amplitude), showing $\mathbf{R}_1$ almost fully captures the observed distortion. Indeed, fitting $\mathbf{R}_1$ alone gives a coefficient of $c_1 = 0.994414$ and reproduces ~98.9% of the observed distortion. This is the RSV which possesses RUM character, hence the RUM is by far the predominant driving structural instability in the parent structure of 4H-$SrMnO_3$. We consider this fitting method to be useful to discriminate the RUM character of structural distortions in other materials which transform as linear combinations of multiple displacive and rotational components.

Table S7. RUM analysis of the Γ_5^- symmetry-mode blocks for the 4H and 6H polytypes. Each potential RUM consists of right-singular vectors (RSVs) whose components delineate the coefficients of linear combinations of displacive (dsp) and rotational (rot) symmetry modes. The relative root-mean-squared deviations (RrmsDs) of each RSV have been provided at the bottom of each column to distinguish their RUM character (RrmsD = 0).

4H polytype				
Mode label	Site symmetry	Irrep	$\mathbf{R}_1$	$\mathbf{R}_2$
Mn1:f:dsp	$3m.$	E	–0.76822	0.64019
Mn1:f:rot	$3m.$	E	–0.64019	–0.76822
		RrmsDs	0.00000	0.76878

6H polytype					
Mode label	Site symmetry	Irrep	$\mathbf{R}_1$	$\mathbf{R}_2$	$\mathbf{R}_3$
Ti1:a:dsp	$\bar{3}m.$	E_u	0.73745	–0.57737	0.35044
Ti2:f:dsp	$3m.$	E	0.52141	0.81648	0.24796
Ti2:f:rot	$3m.$	E	0.42929	0.00014	–0.90317
		RrmsDs	0.21484	1.00000	0.80734

For comparison, we show how this method may be applied to a non-RUM distortion. We use the 6H polytype of $BaTiO_3$, in which the Γ_5^- tilt mode stems from a SOJT distortion. The RSVs for this polytype are shown in Table S7, and the corresponding displacement mode amplitudes and fitting results are shown in Table S9. In this case, the majority of the observed distortion is described by $\mathbf{R}_2$, with the residual being largely weighted towards the Ba displacements (~80% of the residual amplitude). In this case, $\mathbf{R}_2$ only accounts for the displacements of the Ti^{4+} cations, so its large contribution is consistent with how SOJT distortions typically manifest in octahedral environments. However, Table S9 shows that the RSV basis does not fully reproduce the observed distortion, with the residual components for

the O sites being of a similar order of magnitude to those of the observed distortion. This shows that the observed distortion in 6H-$BaTiO_3$ cannot be mapped purely in terms of the degrees of freedom spanned by the RSV basis. Again, this is consistent with how SOJT instabilities typically manifest in octahedral environments, where the Ti and O sites displace in a collinear fashion [7]. Overall, this demonstrates the sensitivity of our fitting approach to discriminate between RUM and non-RUM distortions.

Table S8. Mode amplitudes based on the Γ_5^- RSVs in the 4H polytype predicted by ISOTILT ($\mathbf{R}_n$) compared to those obtained from the refinement against the ID22 data at 10 K ($\mathbf{Q}_{obs}$). Note each RSV has been converted into a purely displacive basis to facilitate comparison to the experimental structure. The $\mathbf{R}_n$ components have been renormalized based on the total amplitude of $\mathbf{Q}_{obs}$ calculated without the Sr(1) component (i.e., Q_{Tot-Sr} = 0.39930 Å), thus ensuring the ISOTILT mode amplitudes are comparable. The mode amplitudes reproduced by fitting against $\mathbf{R}_n$ using Eq. (1) ($\mathbf{Q}_{fit}$) as well as the residual amplitudes ($\Delta\mathbf{Q}$) are also provided.

Site	Symmetry	Irrep	$\mathbf{R}_1$ (Å)	$\mathbf{R}_2$ (Å)	$\mathbf{Q}_{obs}$ (Å)	$\mathbf{Q}_{fit}$ (Å)	$\Delta\mathbf{Q}$(Å)
Sr(1)	$\bar{3}m.$	E_u	–	–	0.09653	0.00000	0.09653
Mn	$3m.$	E	0.12730	0.14905	0.12837	0.14675	-0.01838
O(1)	$.2/m.$	A_u	0.22047	-0.05677	0.22994	0.22474	0.00520
		B_{1u}	-0.22047	0.05677	-0.24076	-0.22474	-0.01602
		B_{2u}	0.15170	-0.25571	0.13036	0.13439	-0.00403
O(2)	$mm2$	B_1	-0.15170	0.25571	-0.12300	-0.13439	0.01139
		c_n	1.04342	0.09343			

Table S9. Mode amplitudes based on the Γ_5^- RSVs in the 6H polytype predicted by ISOTILT ($\mathbf{R}_n$) compared to the Γ_5^- mode amplitudes in 6H-$BaTiO_3$ at 20 K ($\mathbf{Q}_{obs}$). Note each RSV has been converted into a displacive basis to facilitate comparison to the experimental structure. The experimental mode amplitudes for 6H-$BaTiO_3$ have been taken from Ref. [2]. The $\mathbf{R}_n$ components have been renormalized based on the total amplitude of $\mathbf{Q}_{obs}$ calculated without the Ba components (i.e., Q_{Tot-Ba} = 0.29979 Å). The mode amplitudes reproduced by $\mathbf{R}_n$ ($\mathbf{Q}_{fit}$) as well as the residual amplitudes ($\Delta\mathbf{Q}$) are also provided.

Site	Symmetry	Irrep	$\mathbf{R}_1$ (Å)	$\mathbf{R}_2$ (Å)	$\mathbf{R}_3$ (Å)	$\mathbf{Q}_{obs}$ (Å)	$\mathbf{Q}_{fit}$ (Å)	$\Delta\mathbf{Q}$ (Å)
Ba(2)	$3m.$	E_u	–	–	–	0.06846	0.00000	0.06846
Ti(1)	$\bar{3}m.$	E_u	-0.10311	-0.17309	-0.06562	-0.17260	-0.17815	0.00555
Ti(2)	$3m.$	E	-0.07291	0.24477	-0.04640	0.23516	0.23124	0.00392
O(1)	$mm2$	B_1	-0.08522	0.00000	0.23259	0.06133	0.03817	0.02316
O(2)	$.m.$	A'_1	0.17752	0.00000	-0.03365	-0.02592	-0.00685	-0.01908
		A'_2	0.06028	0.00000	-0.16447	0.00114	-0.02699	0.02813
		A"	-0.17752	0.00000	0.03365	-0.01863	0.00685	-0.02548
		c_n	-0.00801	0.97288	0.16116			

Supplementary Note 5. Variable-temperature S-XRD measurements.

We collected additional variable-temperature S-XRD data on the I11 beamline to study the evolution of the crystal structure of 4H-$SrMnO_3$ across the structural and magnetic transitions. High-temperature data were collected using a Cyberstar hot air blower, while low-temperature data were collected using an Oxford Systems Cryostream. High- and low-temperature data were collected continuously using a Mythen position-sensitive detector while heating from 25 °C to 500 °C and 100 K to 300 K, respectively.

In a previous diffraction study [1], the exact transition temperature was noted to be difficult to ascertain due to the gradual suppression of the superstructure reflections near the phase transition. However, it was speculated that the structural transition likely coincided with a magnetization anomaly at T_s = 380 K so that this reflected the real transition temperature. However, our own S-XRD measurements show that T_s exceeds 380 K (Fig. 2b), and fits of a simple Gaussian peak against the (261) superstructure reflection yield an estimate of $T_s \approx 458.4(8)$ K (Figure S7). This temperature is likely slightly overestimated as the peak begins to merge with the background near $T \approx 450$ K. Nevertheless, the FE distortion in this system should persist well above room temperature.

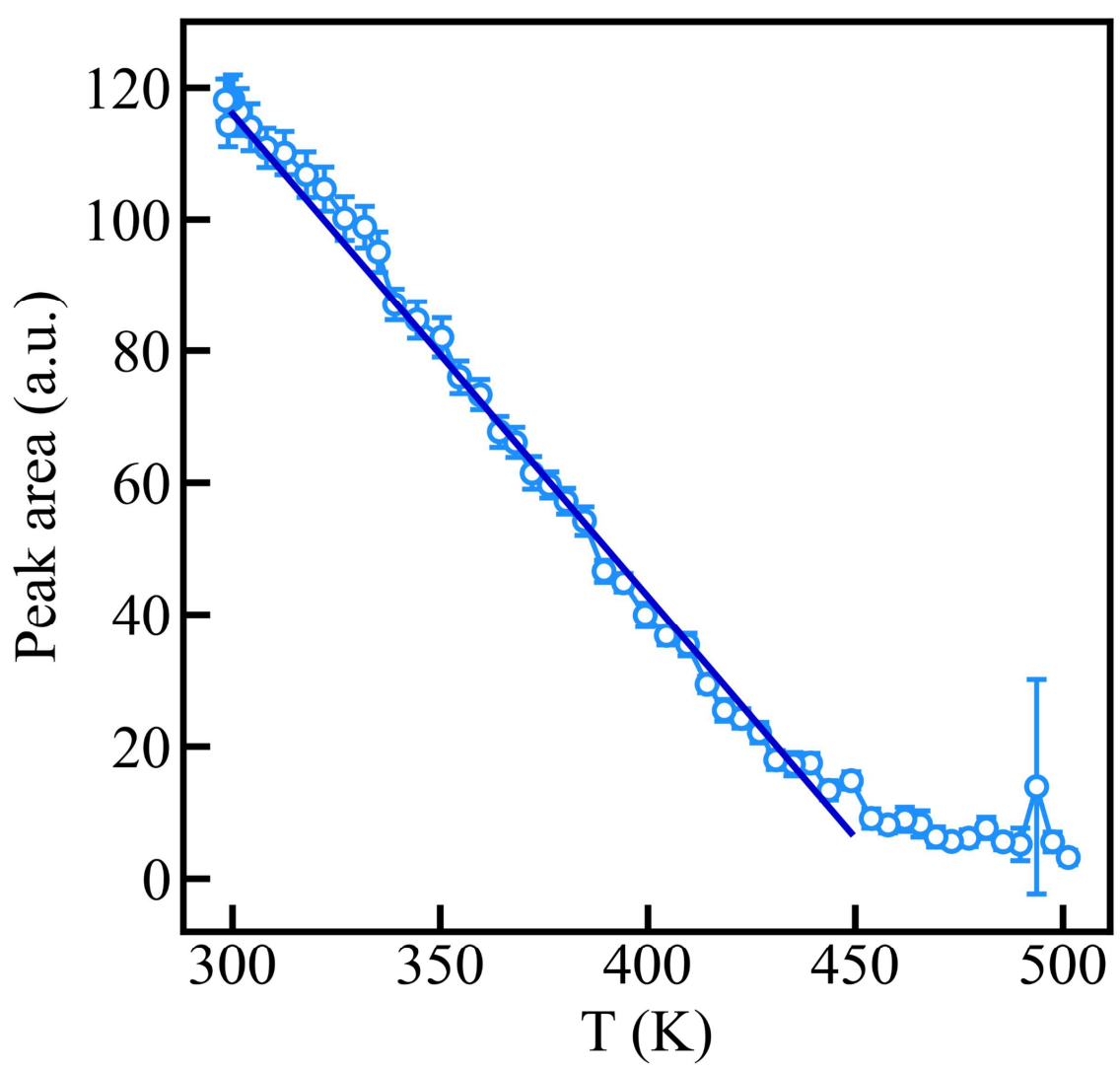

Figure S7. Areas of a Gaussian peak fitted against the (261) superstructure reflection in the $Cmc2_1$ phase of 4H-$SrMnO_3$. A linear regression was used to estimate the transition temperature as 459(1) K.

The fitted lattice parameters show no clear anomalies in the range 100–800 K, exhibiting positive thermal expansion throughout (Figure S8, Figure S9). These fits were based on a $Cmc2_1$ structure first modelled against the 300 K data, before performing sequential refinements against the lower- and higher-temperature datasets. Our recent diffraction study of 6H-$BaTiO_3$ showed its $Cmc2_1 \rightarrow C222_1$ phase transition is marked by a discontinuous change in lattice parameters [2], so the lack of any similar discontinuities in our current diffraction data strongly suggests no analogous structural transition occurs in 4H-$SrMnO_3$. The long-range AFM transition at T_N also does not appear to be accompanied by any

change in structural symmetry, though the volume derivative with respect to temperature reveals the magnetic phase transition is distinguished by a subtle magnetostrictive effect. Additional anomalies in the refined magnitudes of the ferroelastic strain ($\varepsilon(\Gamma_5^+)$) and the Γ_5^- tilts ($Q(\Gamma_5^-)$) corroborate this. As we found for our ID22 data (Supplementary Note 2), the apparent increase in the polar mode amplitudes ($Q(\Gamma_2^-)$) at higher temperatures reflects strong correlations between the refined polar mode components and the anisotropic displacement parameters.

The magnetostriction at T_N does not appear to significantly affect the local Mn^{4+} coordination environments (Figure S10). The intradimer Mn–Mn distances exhibit an overall linear decrease in the range 100–300 K so that the appearance of long-range AFM order does not appear to trigger any notable change in the direct exchange interactions within the face-sharing bioctahedra. The concomitant anomalies in $Q(\Gamma_5^-)$ and $\varepsilon(\Gamma_5^+)$ at T_N suggest the magnetostriction more likely reflects the onset of coupling between the tilts and the AFM/wFM orders, which is consistent with our symmetry predictions. Interestingly, the Mn–O2 distances appear to exhibit anomalous changes in slope at ~240 K. This does not appear to be related to the AFM ordering at T_N, and we detect no other anomalies at this temperature, so further work would be necessary to explore this in more detail.

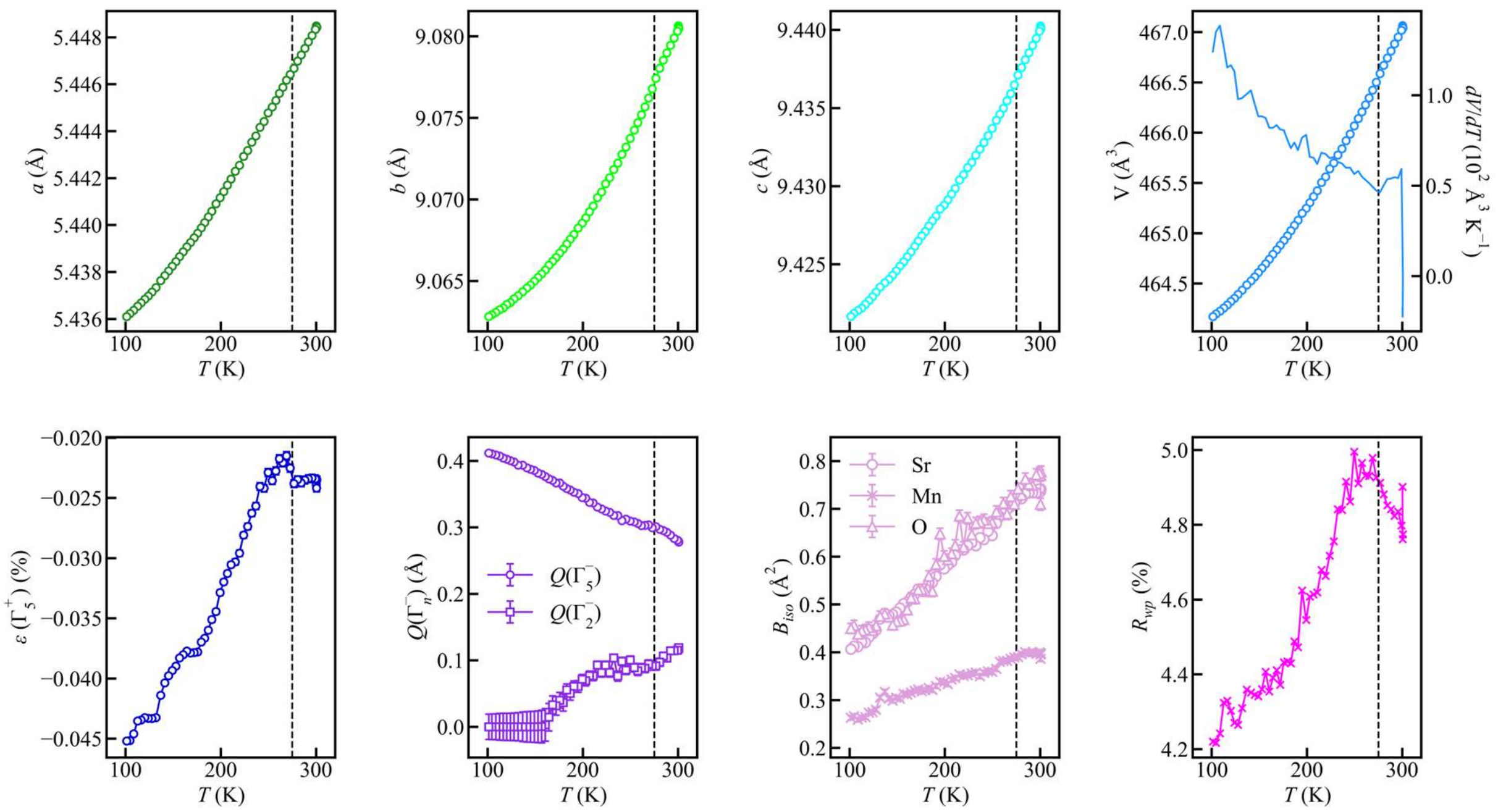

Figure S8. Selected fitting parameters from the $Cmc2_1$ structural model sequentially refined against S-XRD data collected on I11 (Diamond Light Source, U.K.) for 4H-$SrMnO_3$ in the range 100–300 K. The dashed vertical lines denote the AFM transition ($T_N \approx 280$ K). The derivative of V with respect to T has also been shown to highlight a subtle magnetostrictive effect at T_N. The average temperature interval between data points was ~4 K. Where not apparent, the error bars are smaller than the data points.

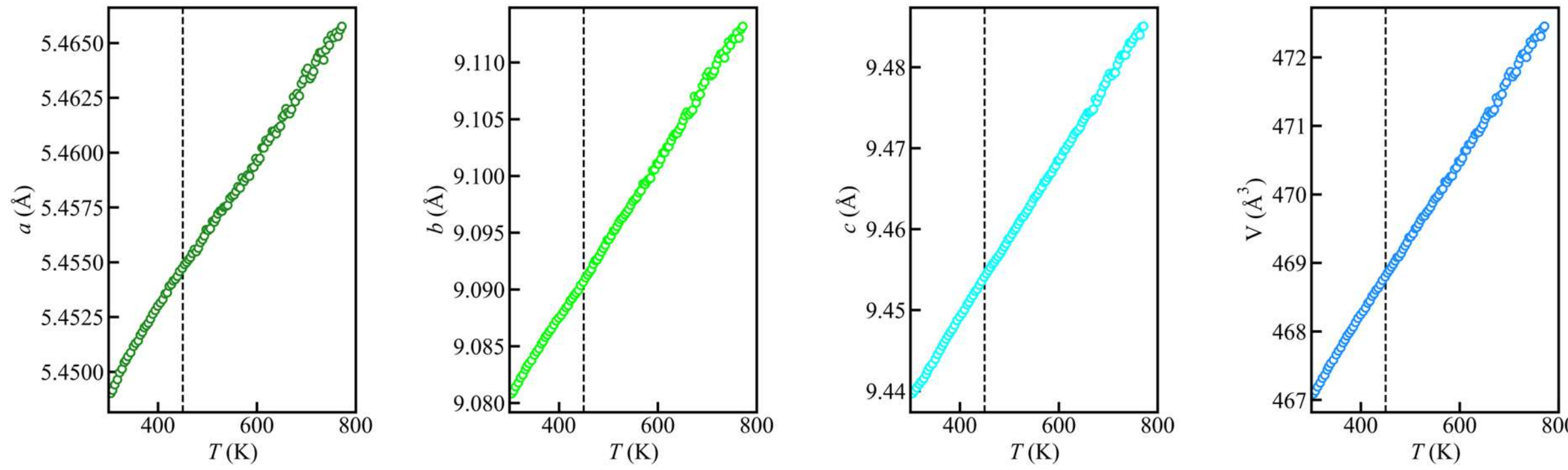

Figure S9. Selected fitting parameters from the $Cmc2_1$ structural model sequentially refined against S-XRD data collected on I11 (Diamond Light Source, U.K.) for 4H-$SrMnO_3$ in the temperature range 300–800 K. The dashed vertical lines denote $T_S \approx 450$ K. As the structure transitions to $P6_3/mmc$ symmetry above T_S, only the variation in lattice parameters has been shown to highlight the lack of any additional phase transitions above room temperature. The average temperature interval between data points was ~4 K. Where not apparent, the error bars are smaller than the data points.

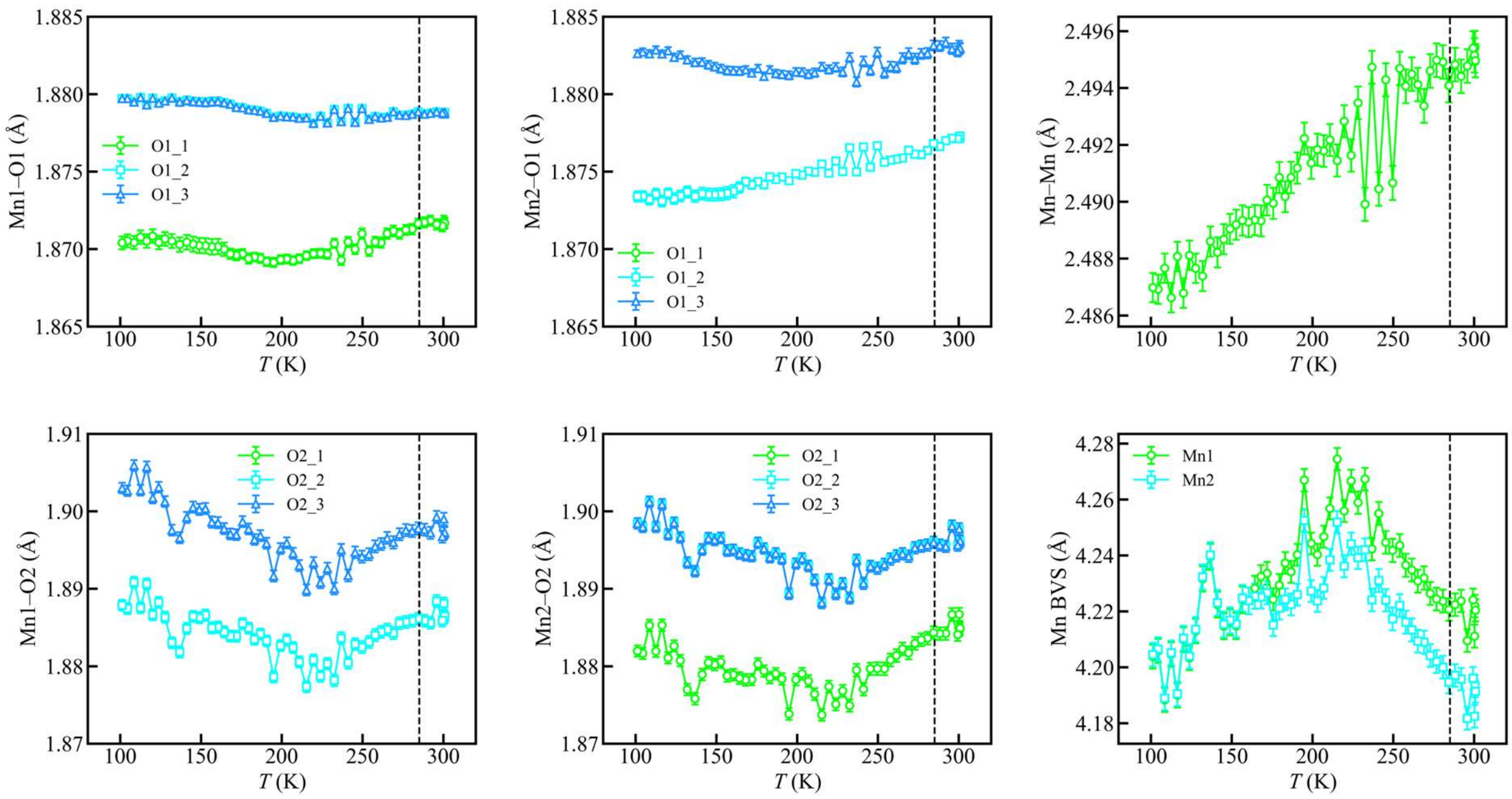

Figure S10. Selected interatomic distances and bond valence sums (BVS) based on sequential refinements of a $Cmc2_1$ model against low-temperature S-XRD data collected at I11 (Diamond Light Source, U.K.) for 4H-$SrMnO_3$. The dashed vertical lines denote the AFM transition ($T_N \approx 280$ K). The O(1) and O(2) sites correspond to the oxygen atoms at the corner- and face-sharing positions, respectively.

Supplementary Note 6. Neutron diffraction measurements and magnetic structure analysis.

To investigate the magnetic structure of 4H-$SrMnO_3$, we performed NPD experiments using the GEM diffractometer at ISIS. Inspection of the room-temperature NPD patterns (Figure S11) revealed no traces of magnetic Bragg peaks, and the $Cmc2_1$ structural model obtained from the S-XRD refinements provided a good fit against these patterns (overall R_{wp} = 5.24%). The Sr, Mn, and O sites could all be refined to within 1% of full occupancy, substantiating that the nominal stoichiometry is accurate (which was also corroborated by our S-XRD refinements). The final refined structural parameters and reliability factors obtained from the multi-bank refinements are provided in Table S10.

Table S10. Crystal and structural parameters extracted from the multi-bank GEM refinement based on data collected at 290 K for 4H-$SrMnO_3$.

4H-$SrMnO_3$, 290 K, SG: $Cmc2_1$, Z = 8				
	a (Å)		5.4503(7)	
	b (Å)		9.4411(1)	
	c (Å)		9.0810(1)	
	V (Å^3)		467.279(12)	
	R_p (%)		5.5711	
	R_{wp} (%)		5.2269	
	GOF		1.56	
Site	x	y	z	B_{iso} (Å^2)
Sr1	0	–0.0048(13)	0	0.37(3)
Sr2	0	0.3309(23)	0.2472(14)	0.37(3)
Mn1	0	0.3385(19)	0.6141(12)	0.06(3)
Mn2	0	0.6716(19)	0.3887(12)	0.06(3)
O1_1	0	0.4941(17)	0.0035(24)	0.41(2)
O1_2	0.2495(23)	0.2621(9)	0.4941(16)	0.41(2)
O2_1	0	0.8207(24)	0.2474(16)	0.41(2)
O2_2	0.7661(21)	0.4068(11)	0.7503(11)	0.41(2)

At 10 K, additional magnetic scattering contributions appear at low Q (see Fig. 3a in the main text). These scattering features can be modelled using the previously reported spin configuration consisting of a layered AFM arrangement [1]. Using ISODISTORT [8,9], we found that this magnetic structure transforms in terms of the parent $P6_3/mmc$ structure as the $m\Gamma_6^-$ irrep. As we are insensitive to the precise OPD in our NPD measurements, we used the (a,0) $m\Gamma_6^-$ OPD obtained from our DFT calculations (Table S5) as our model, corresponding to the Shubnikov group $Cmc'2_1'$. As we are insensitive to the $m\Gamma_6^-$ OPD, we only use this model to refine the overall Mn moment, rather than verify the spatial orientation of the spins. Rietveld fits using this model (Figure S12) yielded a refined Mn moment of 2.34(3) μ_B per Mn at 10 K – this agrees well with the moment of 2.27(1) μ_B obtained in the previous NPD study [1], which is reduced by ~25% from the ordered moment of 3 μ_B expected for high-spin Mn^{4+} due to covalency effects [5]. Full crystallographic details are provided in Table S11.

Table S11. Crystal, structural, and magnetic parameters extracted from the multi-bank GEM refinement based on data collected at 10 K for 4H-$SrMnO_3$.

4H-$SrMnO_3$, 10 K, SG: *Cmc*'2_1', *Z* = 8				
	a (Å)	5.4352(3)		
	b (Å)	9.4237(6)		
	c (Å)	9.0621(1)		
	V (Å^3)	464.157(13)		
	R_p (%)	2.7172		
	R_{wp} (%)	3.4564		
	GOF	1.76		
Site	x	y	z	B_{iso} (Å^2)
Sr1	0	–0.0080(5)	0	0.00(2)
Sr2	0	0.3323(10)	0.2566(14)	0.00(2)
Mn1	0	0.3368(13)	0.6124(17)	–0.01(3)
Mn2	0	0.6766(13)	0.3868(17)	–0.01(3)
O1_1	0	0.4855(10)	0.0139(18)	0.20(2)
O1_2	0.2495(13)	0.2652(5)	0.4993(17)	0.20(2)
O2_1	0	0.8194(11)	0.2416(16)	0.20(2)
O2_2	0.7650(11)	0.4070(7)	0.7551(15)	0.20(2)
Site	M_x	M_y	M_z	M_{total}
Mn1	2.34(3)	0	0	2.34(3)
Mn2	–2.34(3)	0	0	2.34(3)

Unlike the Γ_5^- OPD in our S-XRD experiments, we cannot use the Γ_5^+ ferroelastic strain to infer the mΓ_6^- OPD as there is no unique mapping between the two bases spanned by these irreps (Figure S13). We also tested all possible magnetic space groups generated by the direct sum of the mΓ_6^- and Γ_5^- irreps against our NPD data (Table S12), but we obtained nearly identical residuals in each case (overall R_{wp} ≈ 3.46%). For these reasons, we are insensitive to the precise magnetic space group assignment based on our existing NPD data alone. Nevertheless, we can still deduce the character of the wFM component based on symmetry arguments. Table S12 shows all possible magnetic irreps which are generated through the direct sum of the Γ_5^- and mΓ_6^- irreps. No matter which Γ_5^- and mΓ_6^- OPDs are experimentally adopted, a secondary magnetic component transforming as mΓ_6^+ is always present, and this is permitted by the site symmetries of the 4H polytype to yield a non-zero net moment across the Mn^{4+} sites. None of the other secondary magnetic representations obtained by the direct sums of Γ_5^- and mΓ_6^- correspond to FM orders, and the mΓ_6^- irrep alone is insufficient to yield the mΓ_6^+ component on an improper basis. These symmetry arguments highlight the essential role of the Γ_5^- tilts to induce the wFM component in 4H-$SrMnO_3$. Indeed, this is precisely why no weak FM component emerges at T_N in the untilted Ba analogue 4H-$BaMnO_3$ [10] despite this material sharing the same mΓ_6^- AFM spin configuration in the ground state [11].

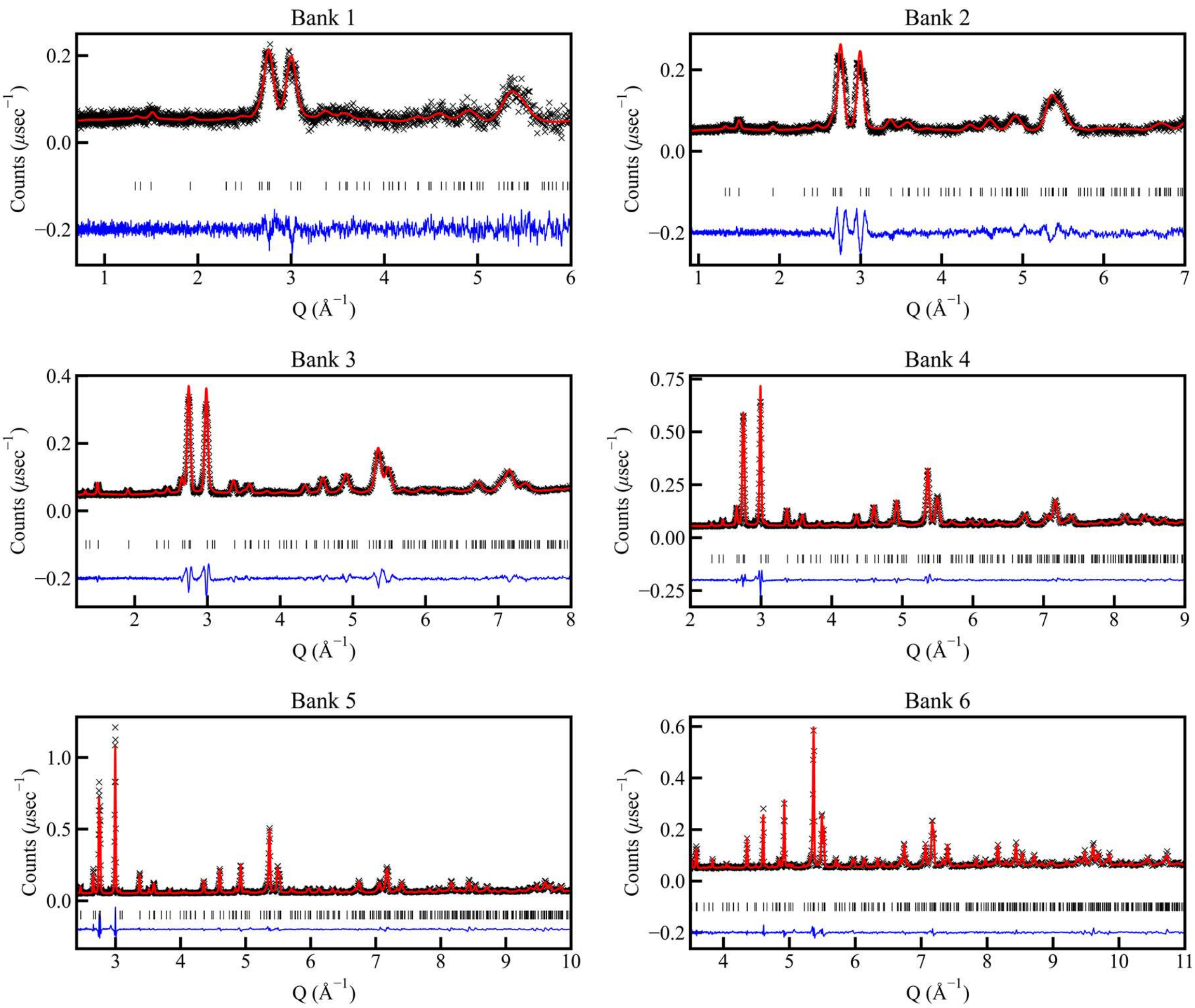

Figure S11. Multi-bank Rietveld fit of the *Cmc*2_1 structural model against room-temperature NPD data collected on GEM for 4H-$SrMnO_3$. Black ticks represent the positions of the nuclear Bragg reflections. Overall R_{wp} = 5.24%.

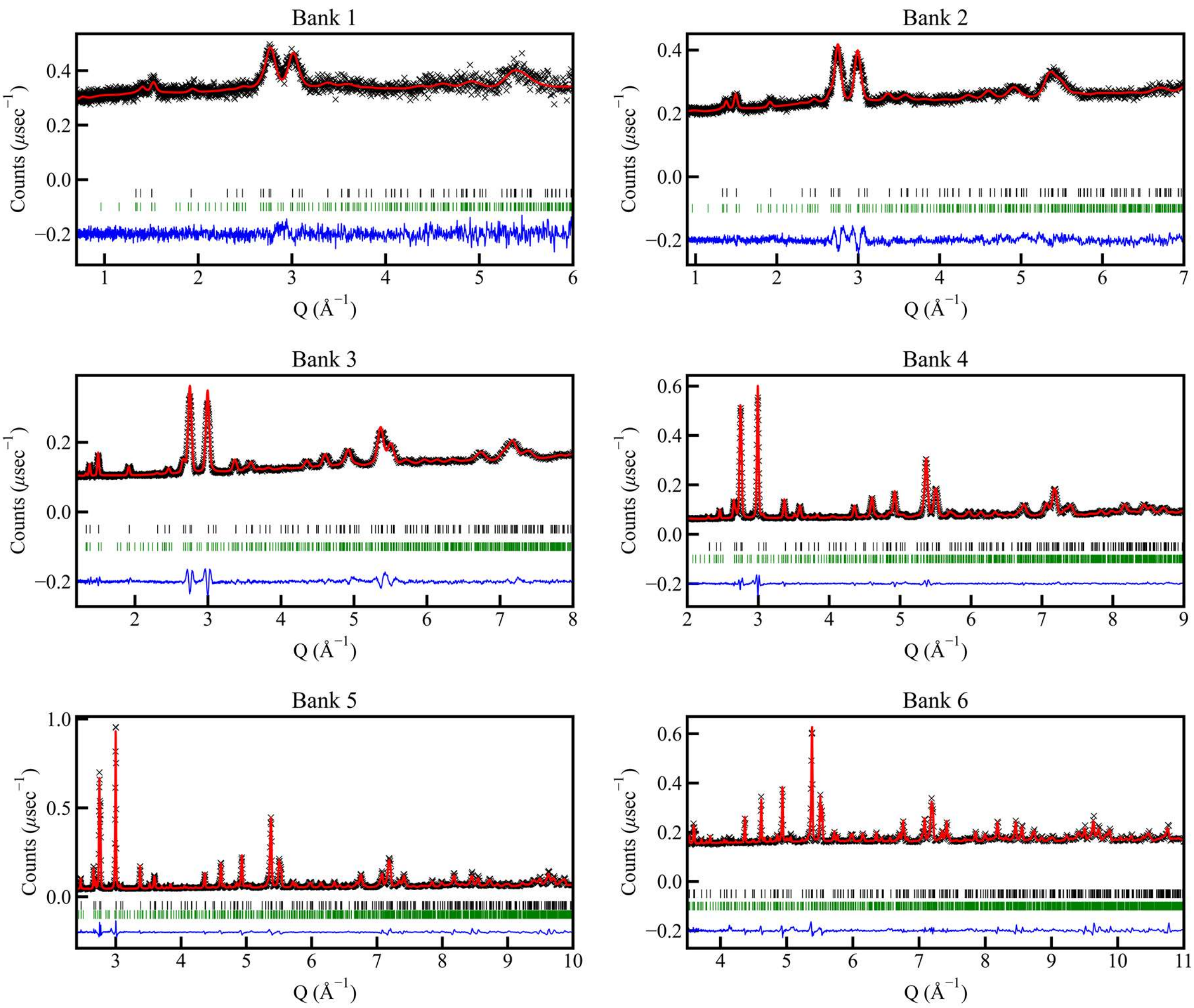

Figure S12. Multi-bank Rietveld fit of the *Cmc*'2_1' magnetic structural model against 10 K NPD data collected on GEM for 4H-$SrMnO_3$. Black and green ticks represent the positions of the nuclear and magnetic Bragg reflections, respectively. Overall R_{wp} = 3.73%.

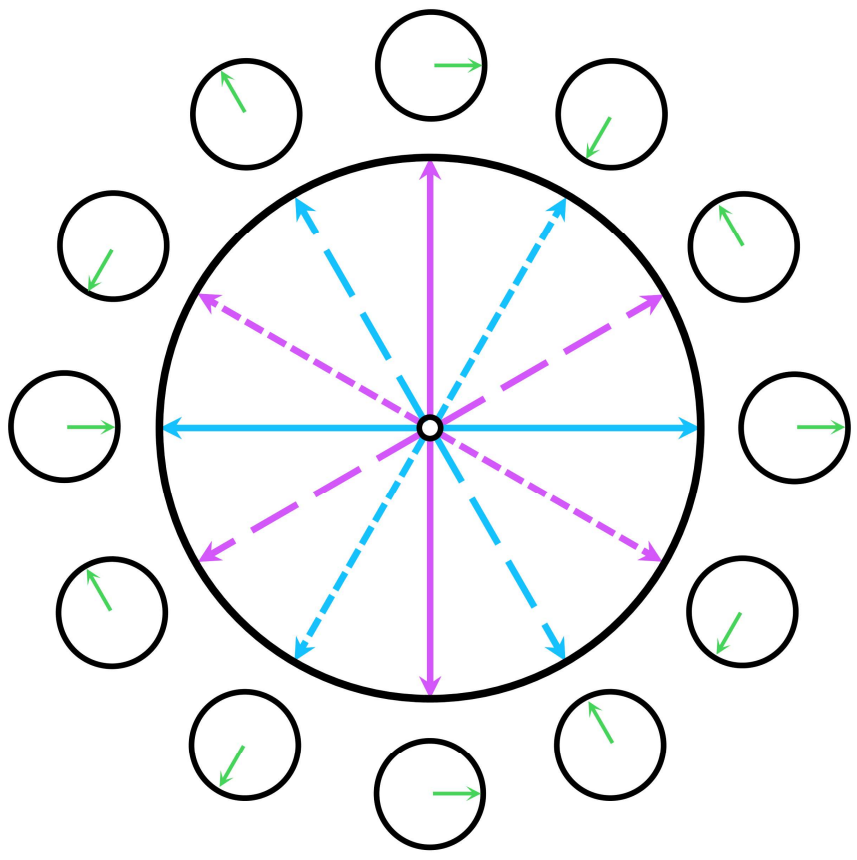

Figure S13. The propagation between the $m\Gamma_6^-$ and Γ_5^+ order parameter spaces, taking $m\Gamma_6^-$ as the primary order parameter. The central circle corresponds to the $m\Gamma_6^-$ space, with blue and magenta arrows denoting the two sets of high-symmetry OPDs corresponding to *Cmc'm* and *Cm'cm* symmetries, respectively. The outer circles show how the choice of $m\Gamma_6^-$ OPD propagates to the Γ_5^+ OPD. Arrows of the same line style in the central circle map to the same strain OPD, highlighting that equivalent strain OPDs may map to distinct $m\Gamma_6^-$ OPDs.

Table S12. All combinations of secondary magnetic irreps based on direct sums of the primary Γ_5^- and $m\Gamma_6^-$ irreps. Note that only the irreps which are permitted by the site symmetries of the 4H polytype to yield a non-zero moment have been shown. The epikernels of the primary irreps as well as the final Shubnikov groups obtained by the direct sums of Γ_5^- and $m\Gamma_6^-$ have been provided next to each OPD for reference. The only representation corresponding to ferromagnetic order is $m\Gamma_6^+$ (highlighted in bold), and this irrep is obtained through all possible direct sums of Γ_5^- and $m\Gamma_6^-$.

	Γ_5^- OPD		
$m\Gamma_6^-$ OPD	(a,0) – $C222_1$	(a, $\sqrt{3}$a/3) – $Cmc2_1$	(a, b) – $P2_1$
(a,0) – *Cmc'm*	$m\Gamma_4^+$, $\mathbf{m\Gamma_6^+}$ ($C22'2_1'$)	$\mathbf{m\Gamma_6^+}$ ($Cmc'2_1'$)	–
(a, $\sqrt{3}$a/3) – *Cm'cm*	$m\Gamma_3^-$, $\mathbf{m\Gamma_6^+}$ ($C22'2_1'$)	$m\Gamma_3^-$, $m\Gamma_4^+$, $\mathbf{m\Gamma_6^+}$ ($Cm'c2_1'$)	–
(a, b) – $P2_1'/m$	–	–	$m\Gamma_3^-$, $m\Gamma_4^+$, $\mathbf{m\Gamma_6^+}$ ($P2_1'$)

Supplementary Note 7. Magnetic property measurements

Figure S14 shows full magnetic susceptibility data for both 4H-$BaMnO_3$ and 4H-$SrMnO_3$. The sample of 4H-$BaMnO_3$ was prepared following the same protocol for 4H-$SrMnO_3$, with the final heating step performed at 1325 °C instead of 1250 °C to avoid the formation of unwanted polytypes [11], and similar to our 4H-$SrMnO_3$ samples, only a trace amount of a Mn_3O_4 impurity phase was formed (~0.4 wt.% by Rietveld refinement). The susceptibility data for 4H-$BaMnO_3$ is broadly consistent with previous reports, with the zero-field-cooled (ZFC) and field-cooled (FC) data largely overlapping until ~45 K. This feature was previously proposed to represent a second magnetic transition involving a canting of the Mn^{4+} spins [11], but our present symmetry insights on 4H-$SrMnO_3$ (namely, that the wFM moment is only generated through coupling to the Γ_5^- tilts) refute this possibility. We note that the spinel-type Mn_3O_4 impurity phase detected in our diffraction experiments also displays a large ZFC-FC divergence below 42 K [12], so we attribute the low-temperature divergence in both samples to this impurity. We do not detect any other magnetic impurities in either our S-XRD or NPD measurements.

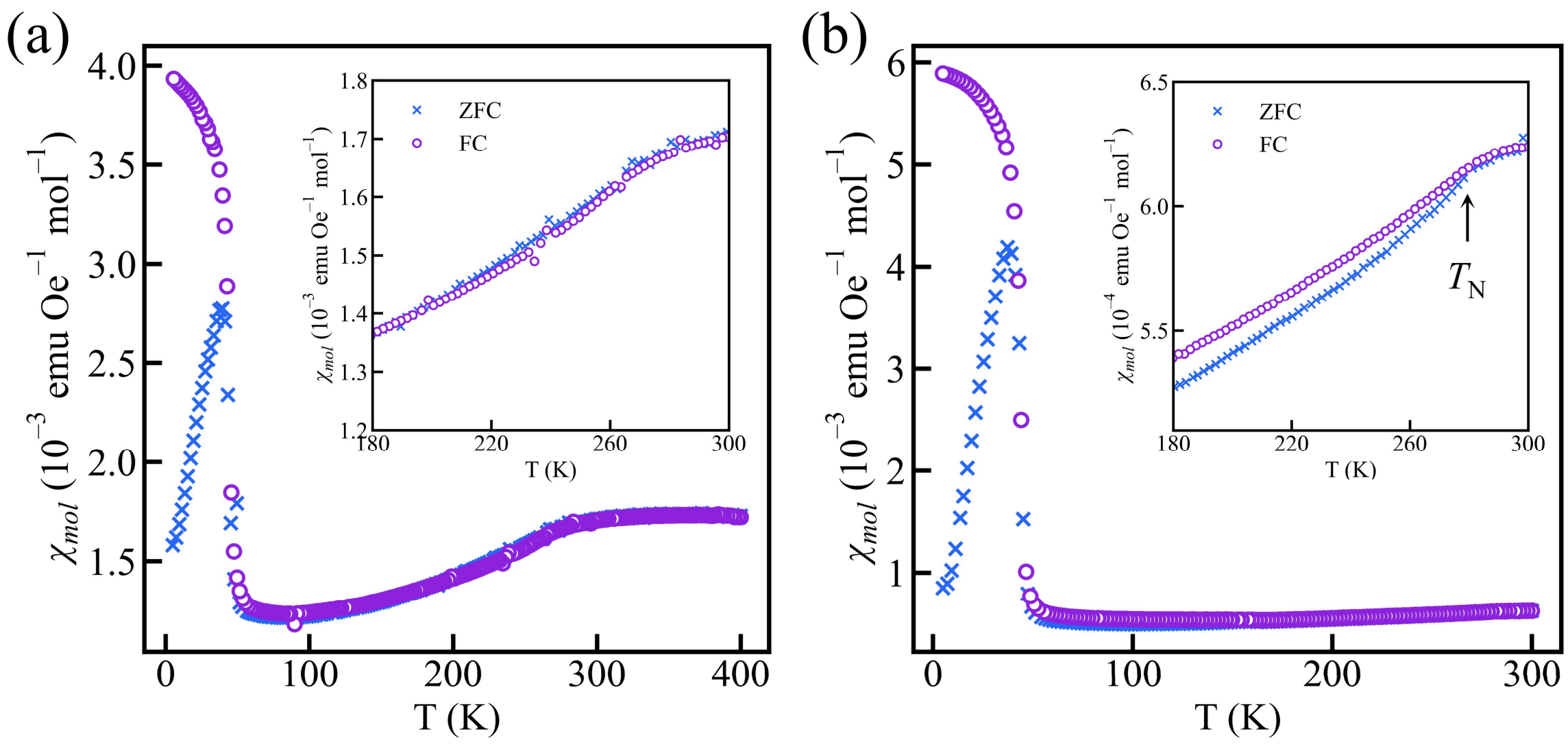

Figure S14. Comparison of the molar magnetic susceptibility (χ_{mol}) for (a) 4H-$BaMnO_3$ and (b) 4H-$SrMnO_3$. The insets expand the region around the Néel temperature (T_N), highlighting the lack of divergence between the ZFC and FC traces for 4H-$BaMnO_3$.

The raw hysteresis loops for 4H-$SrMnO_3$ collected between 200 K and 300 K are shown in Figure S15a. The overall response is nearly linear, though consistent with previous reports [13] the low-field response (–2 kOe < H <2 kOe) is non-linear, suggesting a possible spin-flop transition occurs. This likely also relates to an unusual anomaly detected in our DC resistivity data which was suppressed upon the application of a magnetic field (Supplementary Note 8). Careful inspection of the remanent magnetization (M_r) shows a systematic enhancement in the wFM component below T_N, reflecting coupling between the long-range AFM order and the tilts. A residual M_r persists above T_N, but given

that an extended regime of short-range AFM order was reported to precede T_N [14,15], we conclude that this still reflects the tilt coupling.

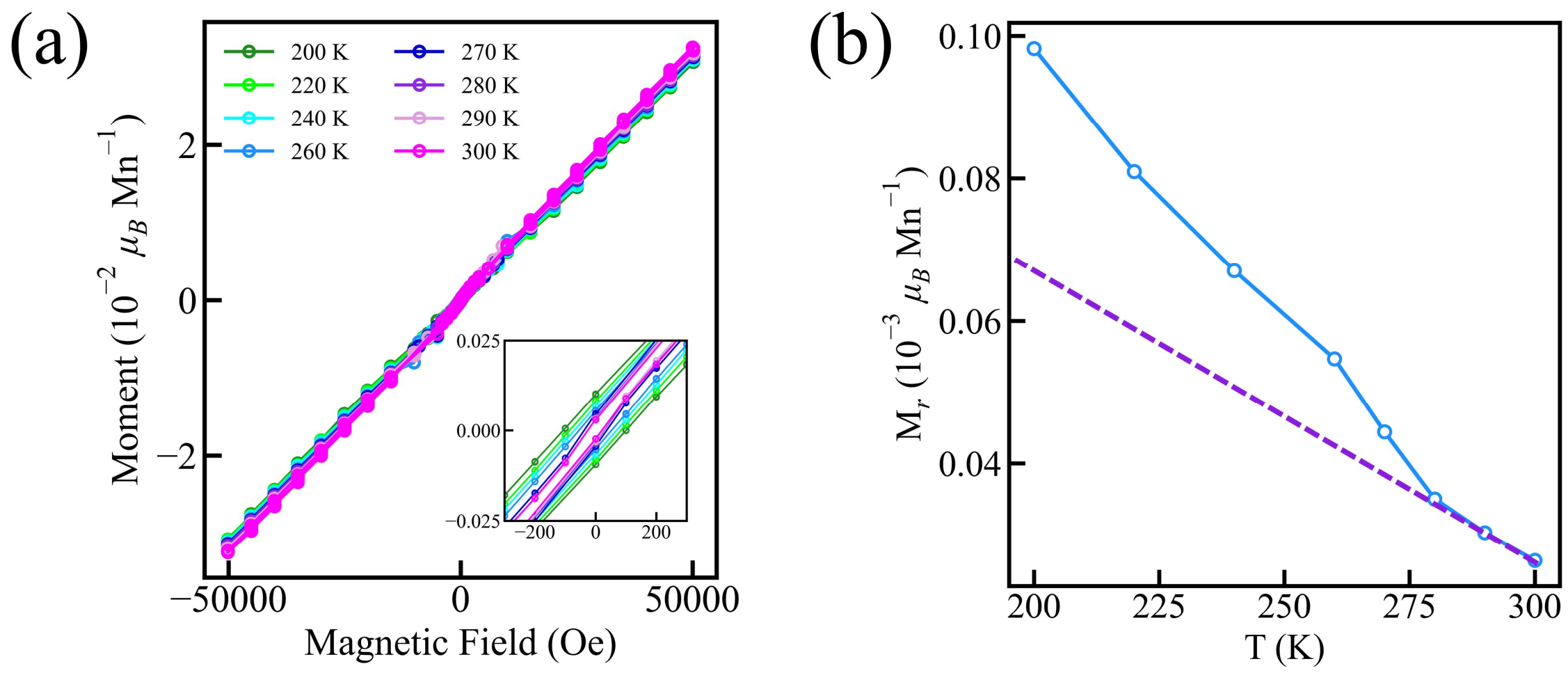

Figure S15. (a) Magnetization versus applied field loops collected between 200 K and 300 K for 4H-$SrMnO_3$. The inset expands the low-field regime to highlight the opening of a wFM component below T_N. (b) The change in remanent magnetization (M_r), highlighting the enhancement below $T_N \approx 280$ K.

Our charged DFT calculations suggested that electron-doped samples of $SrMnO_3$ would allow for the magnitude of the wFM moment to be enhanced. To verify this, we prepared a reduced sample of $SrMnO_{3-x}$ by heating oxygen-stoichiometric $SrMnO_3$ to 1250 °C for 24 h before quenching in air. By comparing the refined lattice parameters (Table S13) to reference literature values [16], the sample stoichiometry can be estimated as $SrMnO_{\sim 2.97}$, corresponding to ~0.06 e^- per Mn. Subsequent DC susceptibility measurements (Figure S16) showed that the magnitude of the wFM moment was enhanced relative to the oxygen-stoichiometric sample, though the enhancement is less significant compared to the 10% Ca-substituted sample. Nevertheless, electron doping presents a promising route to further enhance the wFM moment and stabilize the wFiM state predicted by our DFT calculations.

Table S13. Refined lattice parameters for $SrMnO_{2.97}$ based on laboratory XRD data collected at room temperature.

a (Å)	5.446(1)
b (Å)	9.432(1)
c (Å)	9.095(1)
Volume (Å^3)	234.49(5)

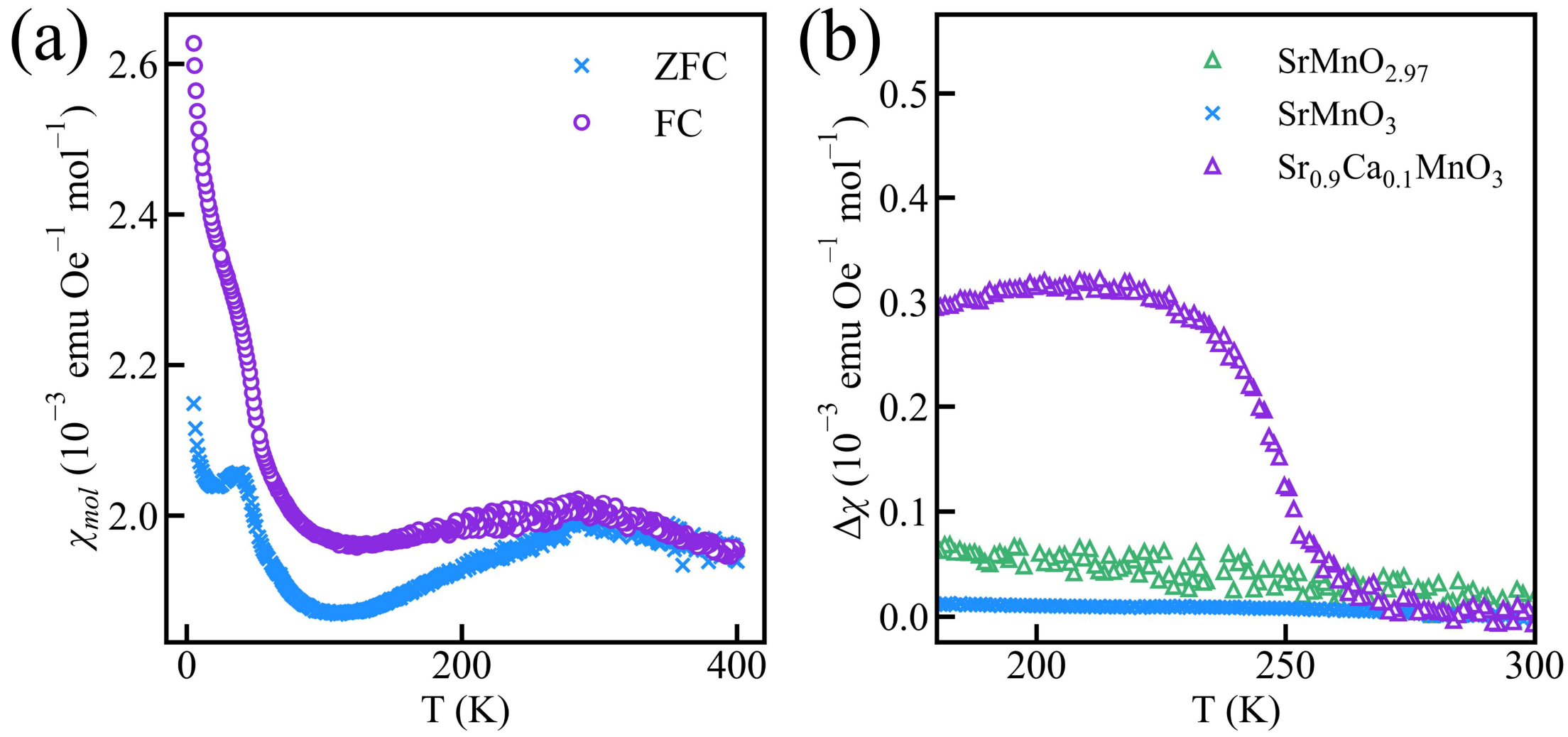

Figure S16. (a) χ_{mol} for 4H-$SrMnO_{2.97}$. The peak near ~45 K is due to a Mn_3O_4 impurity, while a low-temperature Curie tail indicates the presence of a residual paramagnetic component. (b) Comparison of $\Delta\chi_{\mathrm{mol}}$ to $SrMnO_3$ and $Sr_{0.9}Ca_{0.1}MnO_3$, showing that the wFM component is enhanced for the reduced sample, but smaller compared to the 10% Ca-substituted sample.

Supplementary Note 8. DC resistivity and dielectric measurements.

We sought to establish the electrical transport properties of our samples to assess the feasibility of performing switching experiments. Our variable-temperature DC resistivity data (Figure S17a) show that 4H-$SrMnO_3$ behaves as a semiconductor at room temperature, similar to the canonical ME perovskite $BiFeO_3$. However, on cooling to T_N, the resistivity rapidly becomes immeasurably large, suggesting the long-range AFM ordering is linked to an abrupt change in band gap. This is consistent with the AFM insulating ground state predicted by our DFT calculations (Supplementary Note 3). Above 300 K, the transport does not obey a simple Arrhenius dependence (Figure S17b), and neither polaron nor variable-range hopping models yielded good fits against the observed data. Close inspection of the derivative (Figure S18a) suggests this is due to a resistivity anomaly near ~350 K, but no corroborating feature of this appears in our diffraction experiments or our magnetic susceptibility measurements. This also does not appear to be related to the Mn_3O_4 impurity we identified in our diffraction experiments. Interestingly, we find that the anomaly disappears upon the application of a 70 kOe field (Figure S18b), at which point the transport mechanism can be modelled well with a 3D Mott variable-range hopping (VRH) mechanism (Figure S19). This suggests there is a field-induced change in charge transport, which is potentially consistent with the apparent spin-flop transition we observed in our magnetization measurements (Figure S15).

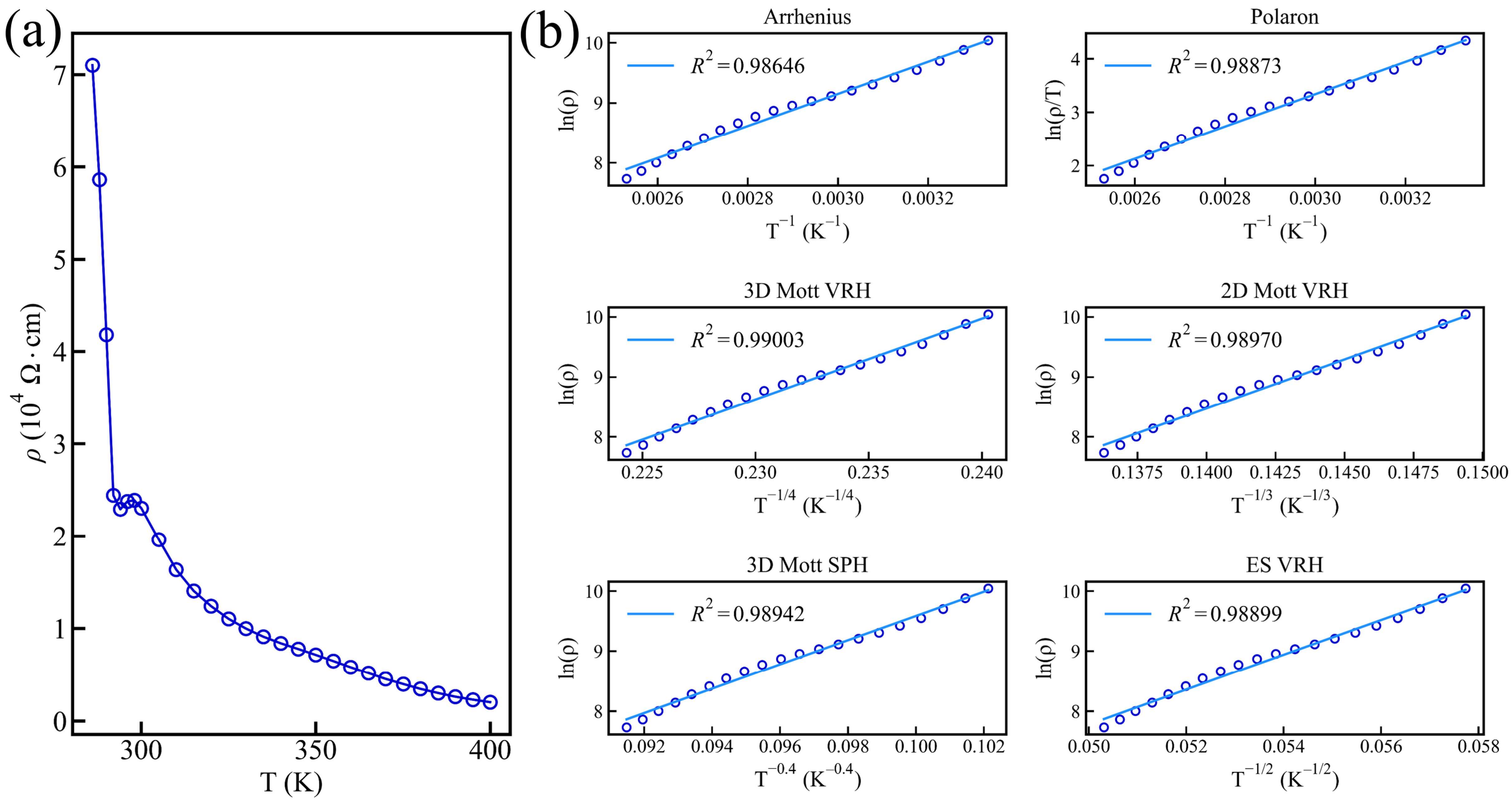

Figure S17. DC resistivity (ρ) measurements on 4H-$SrMnO_3$ performed in the absence of an applied magnetic field. (a) $\rho(T)$ in the range 280–400 K; ρ becomes immeasurably large below 285 K. (b) Comparison of various transport models fitted in the range 300–400 K. "VRH" and "SPH" refer to variable-range hopping and spin polaron hopping, respectively, while "ES VRH" refers to Efros-Shlovskii VRH. The R^2 values have been overlaid for each plot for comparison.

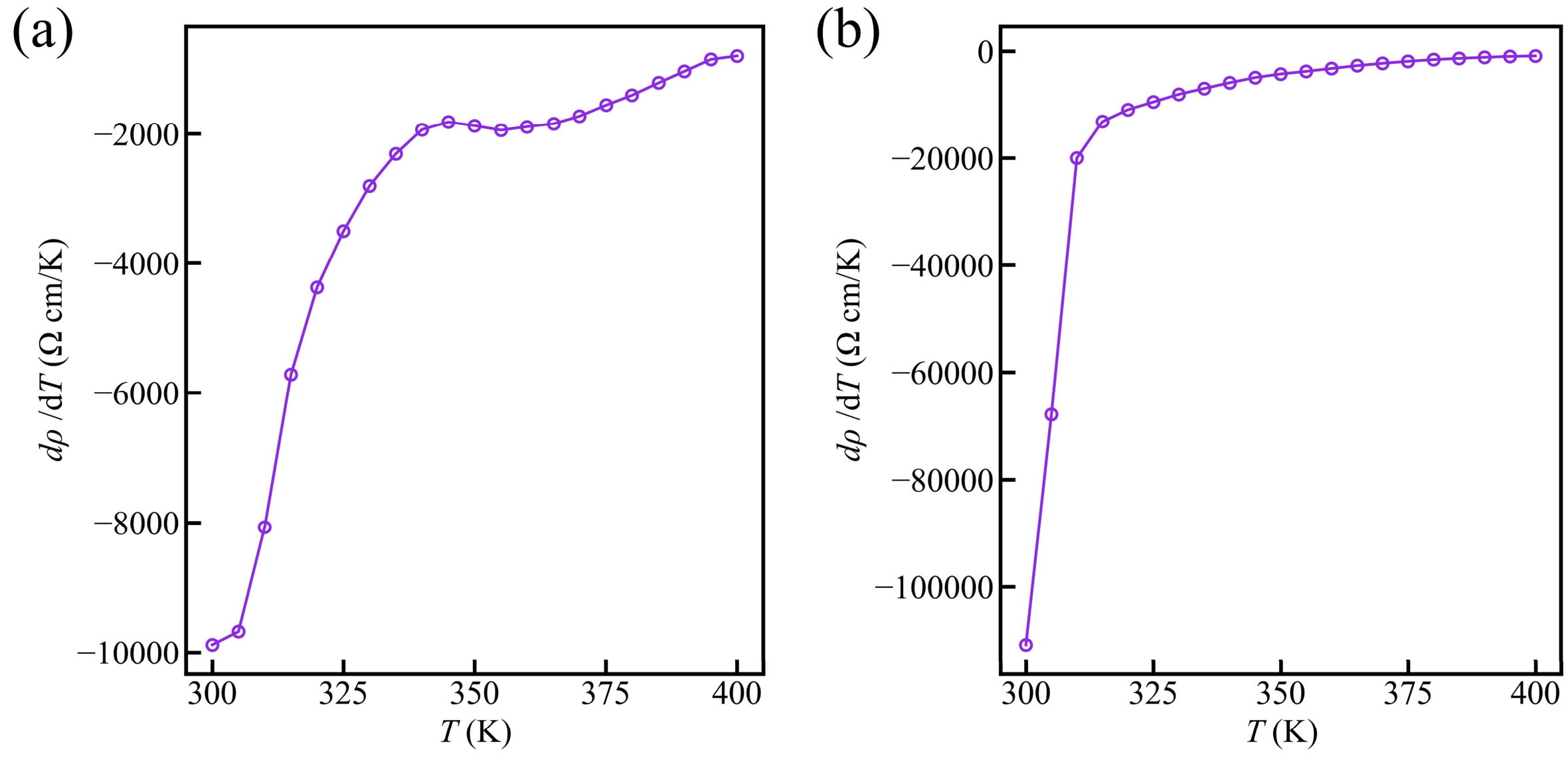

Figure S18. Derivative of ρ with respect to T for the data collected under (a) 0 kOe and (b) 70 kOe, respectively. In both cases, data were collected on cooling.

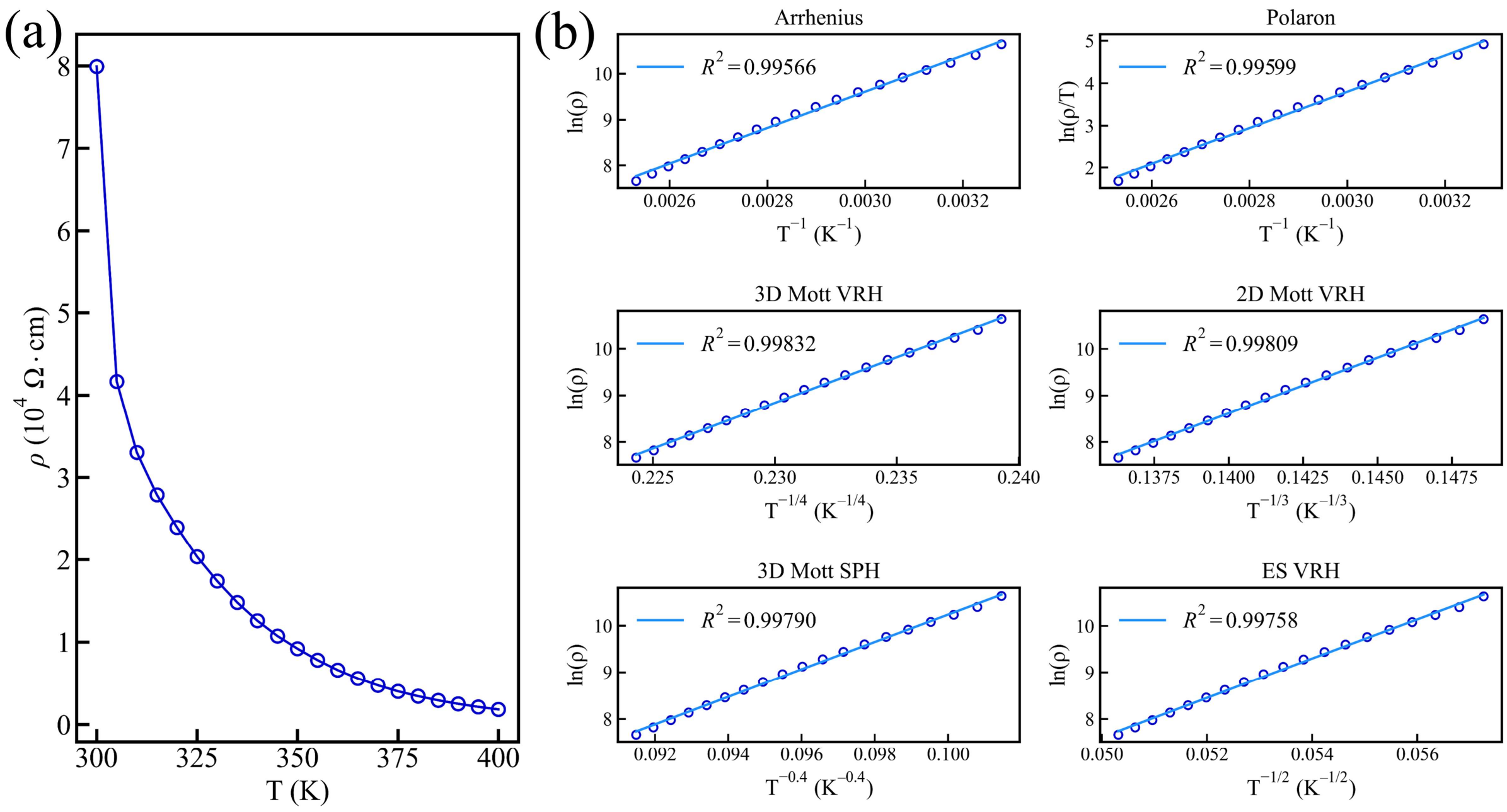

Figure S19. DC resistivity (ρ) measurements on 4H-$SrMnO_3$ performed with a 70 kOe field. (a) $\rho(T)$ in the range 300–400 K; ρ becomes immeasurably large below 300 K. (b) Comparison of various transport models fitted in the range 300–400 K. The R^2 values have been overlaid on each plot for comparison.

The semiconducting nature of our samples makes it challenging for us to obtain reliable dielectric data on our samples and observe ferroelectric switching on an experimental basis. Nevertheless, we attempted to perform dielectric characterization measurements on our polycrystalline samples of 4H-$SrMnO_3$ (Fig. S20). Data were collected from room temperature (RT) to 200 °C at various frequencies

using a 4980 Precision LCR meter (Agilent, Hewlett-Packard, USA). At room temperature, the dielectric loss (tanδ) is already prohibitively large (~15). Such losses prevent us from directly detecting any dielectric feature around the ferroelectric transition at T_S in our own samples, but polarization switching has been achieved recently in 4H-$SrMnO_3$ at room temperature with a maximum polarization of ~0.03 µC cm^{-2}. [17] This is in excellent agreement with the magnitude of polarization obtained in our present DFT calculations (Supplementary Note 3), granting us confidence that our crystallographic and symmetry-informed insights are correct.

Closer to T_N (~280 K, ~7 °C), we identify signs of a peak in the dielectric constant at low frequencies (Fig. S20) to indicate coupling between the electrical and magnetic degrees of freedom. However, the strong dielectric loss and frequency dispersion suggests there are still substantial contributions from conductivity-related polarization mechanisms. Consequently, our present dielectric data alone do not permit an unambiguous assignment of the incipient dielectric feature to any magnetoelectric coupling. Nevertheless, the presence of such a feature would appear to be consistent with previously reported dielectric measurements on lower-conductivity samples, where broad peaks in the dielectric constant and loss have been observed near T_N. [13] This broadening is potentially reflective of the extended regime of short-range AFM ordering that is thought to arise above T_N [15], which may make it difficult to observe any sharp dielectric feature near this temperature. Further work should seek to further suppress the conductivity of these 4H-$SrMnO_3$ ceramics and perform more extensive dielectric characterization measurements. Ceramic engineering strategies, similar to what has been achieved in $BiFeO_3$-based ceramics [18,19], will likely be key in this respect.

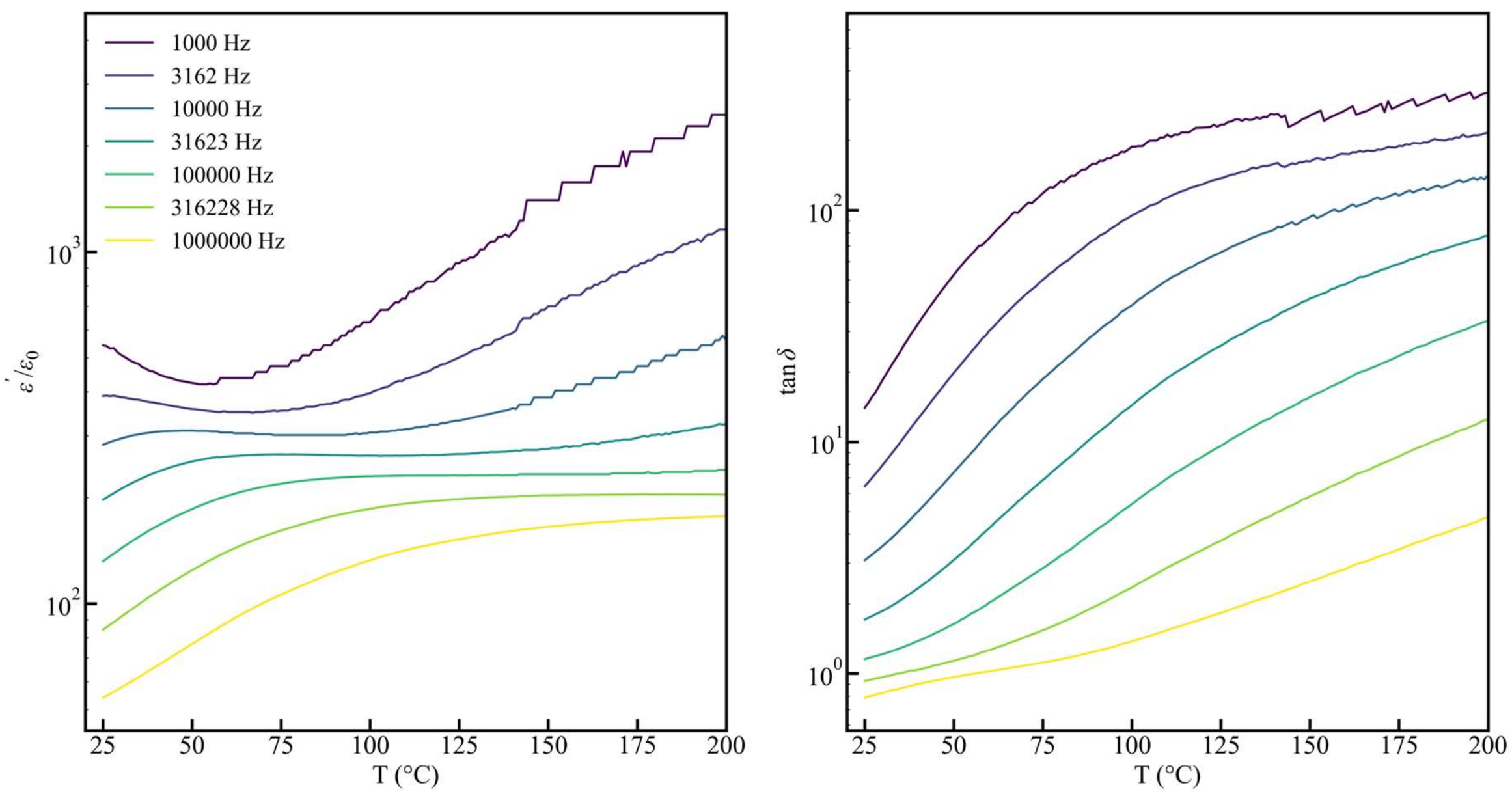

Figure S20. Temperature-dependent dielectric constant ($\varepsilon'/\varepsilon_0$, left) and dielectric loss (tanδ, right) of 4H-$SrMnO_3$.

Supplementary Note 9. Compositional control over the wFM moment.

Our magnetic susceptibility data indicate that the increase in wFM moment for the Ca-substituted samples is associated with enhanced tilting of the Mn_2O_9 bioctahedra. To demonstrate this explicitly, we measured high-resolution S-XRD data at ID22 on the x = 0.05 and 0.10 Ca-substituted samples at 200 K, where the divergence between ZFC and FC susceptibility ($\Delta\chi_{200K}$) can be reliably distinguished. Rietveld refinement of the same *Cmc*2$_1$ structural model used for the 4H-$SrMnO_3$ parent phase revealed that $\Delta\chi_{200K}$ increases monotonically with $Q(\Gamma_5^-)$ (Fig. S21). As such, the wFM component can be systematically enhanced by modifying the amplitude of the Γ_5^- tilts through chemical pressure effects. We anticipate that similar enhancements could be achieved through physical pressure, including the application of hydrostatic pressure or epitaxial strain.

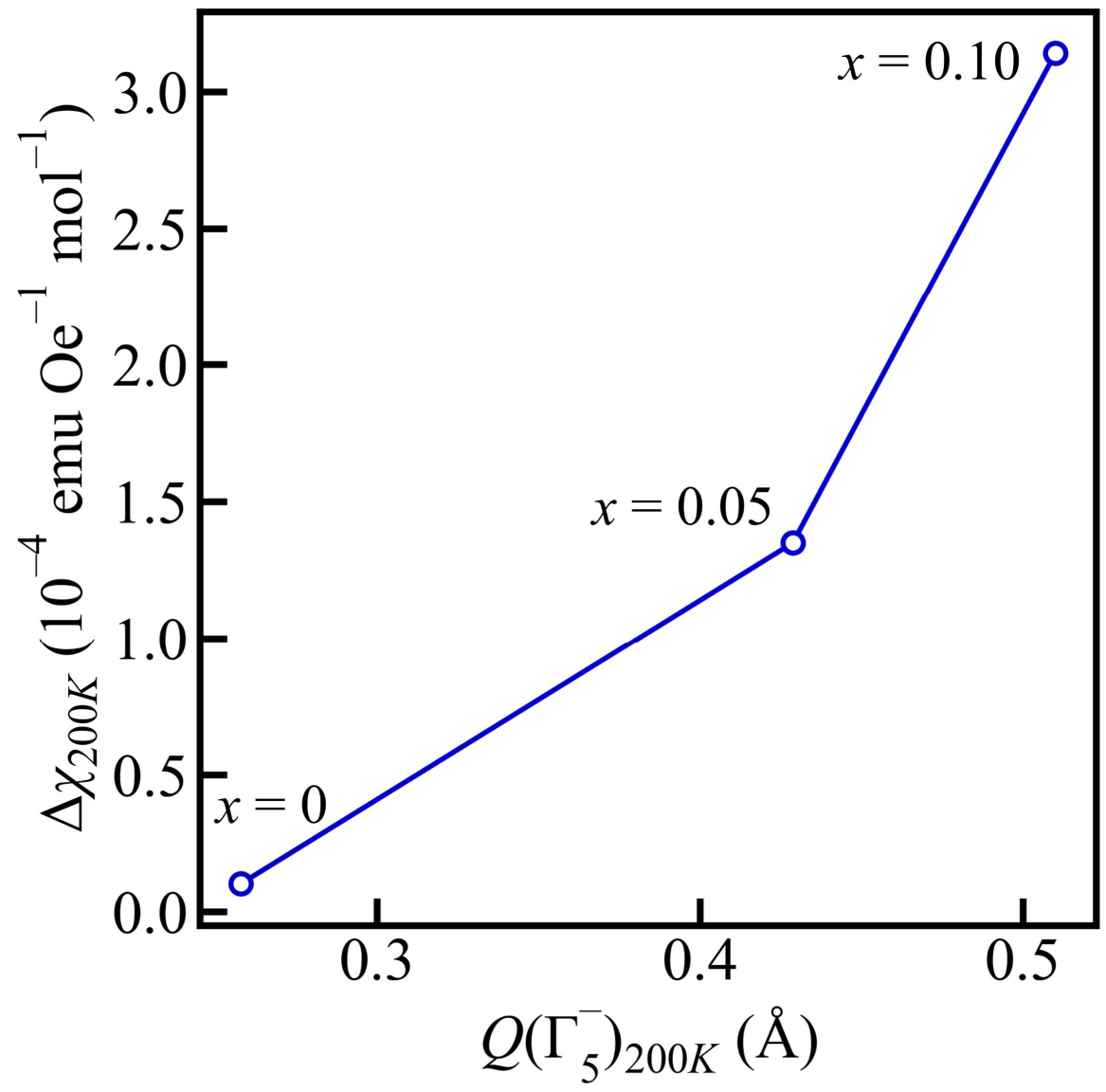

Figure S21. Correlation between $\Delta\chi_{mol}$ and $Q(\Gamma_5^-)$ at 200 K based on Rietveld fits against the *Cmc*2$_1$ structural model for 4H-$Sr_{1-x}Ca_xMnO_3$ (x = 0, 0.05, and 0.10).

Supplementary Note 10. Landau free energy analysis.

The nature of the coupling between the order parameters in 4H-$SrMnO_3$ can be determined by analyzing the symmetry-invariant polynomials in the Landau free energy expansion of the system. For this, we treat the Γ_5^- (a,b) tilts, the Γ_2^- (c) polar mode, the mΓ_6^- (d,e) AFM component, and the mΓ_6^+ (f,g) wFM component as the salient order parameters; for convenience, we rewrite the 2D order parameters in polar form, leading to (ρ, φ) for Γ_5^- and (M_1, θ_1), (M_2, θ_2) for mΓ_6^- and mΓ_6^+, respectively. We also rewrite Γ_2^- (c) as P, taking the magnitude of any spontaneous polarization to be proportional to the atomic displacements which transform under this irrep. For simplicity, we neglect the effects of the Γ_5^+ ferroelastic strain as its magnitude is essentially negligible in the ground state (Figure S4), and we also neglect contributions from any of the other secondary order parameters.

In our model, we consider terms up to the sixth order of the free energy expansion so as to account for the angular anisotropy of the Γ_5^-, mΓ_6^-, and mΓ_6^+ terms. The full free energy expansion according to these conditions is extremely complex, so it has been provided alongside the Supplementary Material. For the purposes of our discussion, we instead consider a minimal model which considers only the lowest-order coupling terms between the salient order parameters. Although isotropic invariants such as those found in the biquadratic terms $F \sim \gamma_1\rho^2P^2$ or $F \sim \gamma_2 M_1{}^2 M_2{}^2$ will be essential to the overall stability of the low-symmetry phases, we have purposefully omitted these terms to ensure our model only encompasses the minimum number of terms necessary to encapsulate the overall form of the energy landscape. Our minimal model is thus as follows:

$$
\begin{gathered}
F = \alpha_1\rho^2 + \alpha_2\rho^4 + \alpha_3\rho^6 + \alpha_4\rho^6(10 + \cos 6\varphi) \\
+ \alpha_5 P^2 + \alpha_6 M_1^2 + \alpha_7 M_1^4 + \alpha_8 M_2^2 \\
+ \beta_1 P\rho^3 \sin 3\varphi + \beta_2 P M_1 M_2 \sin\Delta + \beta_3 \rho M_1 M_2 \sin\Theta
\end{gathered}
$$

where $\Delta = \theta_1 - \theta_2$ and $\Theta = \theta_1 + \theta_2 - \varphi + \pi/6$. Given that the Γ_5^- tilt and mΓ_6^- AFM modes are unstable with respect to the parent paramagnetic structure, we take $\alpha_1, \alpha_6 < 0$ and include their higher-order terms to maintain boundedness. We also include the sixth-order terms in ρ to account for the anisotropy of the tilts, which dictates the relative stability of the $C222_1$ ($\alpha_4 < 0$, $\varphi = n\pi/3$) and $Cmc2_1$ ($\alpha_4 > 0$, $\varphi = (2n + 1)\pi/6$) structural phases. Analogous anisotropy terms are also present for the mΓ_6^- AFM and mΓ_6^+ wFM orders, but we assume their contribution to the overall energy landscape is negligible compared to the structural order parameters.

First, we consider the coupling between the tilts and the polarization, which is of the form:

$$
F \sim \beta_1 P\rho^3 \sin 3\varphi
$$

The linear coupling in P ensures it may adopt either a positive or negative sign so as to minimize F, hence this can always favor the $Cmc2_1$ phase ($\varphi = (2n+1)\pi/6$) regardless of the sign of the β_1 coefficient.

Symmetry-related $Cmc2_1$ domains are related by $\Delta\varphi = \pm n\pi/3$ (see Figure S1); for odd values of n, the direction of P necessarily flips by 180° to maintain invariance overall. This represents the most likely basis for polarization reversal in 4H-$SrMnO_3$, similar to what has been proposed in hexagonal rare-earth manganites such as $YMnO_3$ [20].

Next, we consider the trilinear coupling between the tilts, the AFM order, and the wFM order, which takes the form:

$$F \sim \beta_2 P M_1 M_2 \sin\Delta, \qquad \Delta = \theta_1 - \theta_2$$

Again, the linear nature of the coupling in P ensures it may adopt either a positive or negative sign so as to minimize F. The transformation $P \rightarrow -P$ necessarily induces $\Delta \rightarrow \Delta \pm \pi$ to maintain invariance. This can occur via the rotation of either θ_1 or θ_2 by 180°, but since the switching barriers are likely to be much higher for θ_1, reorienting θ_2 is far more likely. Nevertheless, this simple picture neglects the role of the coupling to the tilts, which must also reorient as part of any proposed switching mechanism. To consider this on an explicit basis, we also examine the additional trilinear invariant:

$$F = \beta_3 \rho M_1 M_2 \sin\Theta, \qquad \Theta = \theta_1 + \theta_2 - \varphi + \frac{\pi}{6}$$

Here, the 2D anisotropies of the tilts and the AFM/wFM components have been absorbed into the single phase angle Θ. Minimizing F with respect to Θ leads to the condition $\theta_1 + \theta_2 = \varphi - \pi/6$, showing how the orientation of the magnetic order parameters depends upon the orientation of the Γ_5^- tilts. A reversal in P may effect the transformation $\varphi \rightarrow \varphi \pm \pi$. Given F is also minimized for $\Delta = \pm\pi/2$, this triggers a 180° reorientation in either θ_1 or θ_2. Again, θ_2 is much more likely to reorient based on the anticipated switching barriers for the AFM and wFM moments. Verifying the feasibility of this proposed switching mechanism would require a bespoke computational study assessing the relative switching barriers associated with $\Delta\varphi = n\pi/3$, which lies far beyond the scope of the present work. Nevertheless, 4H-$SrMnO_3$ possesses the capacity for ME switching, and further work should seek to explore different switching pathways to facilitate its experimental realization.

Supplementary References

1. Daoud-Aladine, A. *et al.* Structural phase transition and magnetism in hexagonal $SrMnO_3$ by magnetization measurements and by electron, x-ray, and neutron diffraction studies. *Phys. Rev. B* **75**, 104417 (2007).
2. Simpson, S. *et al.* Goldstone-Mediated Polar Instability in Hexagonal Barium Titanate. *Phys. Rev. Lett.* **136**, 116101 (2026).
3. Simpson, S. & Senn, M. S. Octahedral Tilting in Perovskite Polytypes. *Chem. Mater.* **37**, 4524–4533 (2025).
4. Kimura, T. *et al.* Magnetic control of ferroelectric polarization. *Nature* **426**, 55–58 (2003).
5. Søndenå, R., Ravindran, P., Stølen, S., Grande, T. & Hanfland, M. Electronic structure and magnetic properties of cubic and hexagonal $SrMnO_3$. *Phys. Rev. B* **74**, 144102 (2006).
6. Campbell, B. J., Stokes, H. T., Averett, T. B., Machlus, S. & Yost, C. J. The ISOTILT software for discovering cooperative rigid-unit rotations in networks of interconnected rigid units. *J. Appl. Cryst.* **54**, 1847–1856 (2021).
7. Kwei, G. H., Lawson, A. C., Billinge, S. J. L. & Cheong, S. W. Structures of the ferroelectric phases of barium titanate. *J. Phys. Chem.* **97**, 2368–2377 (1993).
8. Stokes, H. T., Hatch, D. M. & Campbell, B. J. ISODISTORT, ISOTROPY Software Suite. iso.byu.edu.
9. Campbell, B. J., Stokes, H. T., Tanner, D. E. & Hatch, D. M. ISODISPLACE: A web-based tool for exploring structural distortions. *J. Appl. Cryst.* **39**, 607–614 (2006).
10. Qin, S. *et al.* High-Pressure Synthesis and Magnetism of the 4H-$BaMnO_3$ Single Crystal and Its 6H-Type Polymorph. *Inorg. Chem.* **60**, 16308–16315 (2021).
11. Adkin, J. J. & Hayward, M. A. Structure and magnetism of 4H-$BaMnO_{3-x}$ ($0\leqslant x\leqslant 0.35$) and 4H-$Ba_{0.5}Sr_{0.5}MnO_{3-x}$ ($0\leqslant x\leqslant 0.21$). *J. Solid State Chem.* **179**, 70–76 (2006).
12. Dwight, K. & Menyuk, N. Magnetic Properties of Mn_3O_4 and the Canted Spin Problem. *Phys. Rev.* **119**, 1470–1479 (1960).

13. Aich, P. *et al.* Fluorinated hexagonal 4H $SrMnO_3$: a locally disordered manganite. *J. Mater. Chem. C* **7**, 3560–3568 (2019).

14. Chamberland, B. L., Sleight, A. W. & Weiher, J. F. Preparation and Characterization of $BaMnO_3$ and $SrMnO_3$ Polytypes. *J. Solid State Chem.* **1**, 506–511 (1970).

15. Battle, P. D., Gibb, T. C. & Jones, C. W. The structural and magnetic properties of $SrMnO_3$: A reinvestigation. *J. Solid State Chem.* **74**, 60–66 (1988).

16. Negas, T. & Roth, R. S. The system $SrMnO_{3-x}$. *J. Solid State Chem.* **1**, 409–418 (1970).

17. Isaza-Zapata, V. *et al.* Structural aspects and magnetoelectric behavior of hexagonal $Sr_{1-x}Ba_xMnO_3$ (x=0, 0.4) manganites: Effect of leakage currents. *Phys. Lett. A* **433**, 128019 (2022).

18. Kalantari, K. *et al.* Ti-Doping to Reduce Conductivity in Bi0.85Nd0.15FeO3 Ceramics. *Advanced Functional Materials* **21**, 3737–3743 (2011).

19. Reaney, I. M. *et al.* Defect chemistry of Ti-doped antiferroelectric Bi0.85Nd0.15FeO3. *Appl. Phys. Lett.* **100**, 182902 (2012).

20. Artyukhin, S., Delaney, K. T., Spaldin, N. A. & Mostovoy, M. Landau theory of topological defects in multiferroic hexagonal manganites. *Nat. Mater.* **13**, 42–49 (2014).
